# Structural Diversity and Highly Specific Host-Pathogen Transcriptional Regulation of Defensin Genes Is Revealed in Tomato

**DOI:** 10.3390/ijms21249380

**Published:** 2020-12-09

**Authors:** Nikolaos Nikoloudakis, Polyxeni Pappi, Emmanouil A. Markakis, Spyridoula N. Charova, Dimitrios Fanourakis, Konstantinos Paschalidis, Costas Delis, Emmanuel A. Tzortzakakis, Georgios Tsaniklidis

**Affiliations:** 1 Department of Agricultural Science, Biotechnology and Food Science, Cyprus University of Technology, Limassol 3036, Cyprus; 2 Department of Viticulture, Vegetable Crops, Floriculture and Plant Protection, Institute of Olive Tree, Subtropical Crops and Viticulture, Hellenic Agricultural Organization ELGO-DIMITRA, Mesa Katsabas, 71307 Heraklion, Crete, Greece; polyxeni.pappi@nagref-her.gr (P.P.); markakis@nagref-her.gr (E.A.M.); etzortza@nagref.gr (E.A.T.); 3 Institute of Molecular Biology and Biotechnology, Foundation for Research and Technology-Hellas (IMBB-FORTH), 70013 Heraklion, Crete, Greece; charova@imbb.forth.gr; 4 Department of Biology, University of Crete, 70013 Heraklion, Crete, Greece; 5 Giannakakis SA, Export Fruits and Vegetables, 70200 Tympaki, Crete, Greece; dimitrios.fanourakis82@gmail.com; 6 School of Agricultural Sciences, Hellenic Mediterranean University, Estavromenos, 71004 Heraklion, Crete, Greece; kpaschal@hmu.gr; 7 Department of Agricultural Technology, School of Agricultural Technology and Food Technology and Nutrition, University of Peloponnese, 24100 Antikalamos, Kalamata, Greece; delis@us.uop.gr

**Keywords:** biotic stress, CMV, cold stress, defensins, *Meloidogyne javanica*, nematodes, PVY, tomato, Verticillium wilt

## Abstract

Defensins are small and rather ubiquitous cysteine-rich anti-microbial peptides. These proteins may act against pathogenic microorganisms either directly (by binding and disrupting membranes) or indirectly (as signaling molecules that participate in the organization of the cellular defense). Even though defensins are widespread across eukaryotes, still, extensive nucleotide and amino acid dissimilarities hamper the elucidation of their response to stimuli and mode of function. In the current study, we screened the *Solanum lycopersicum* genome for the identification of defensin genes, predicted the relating protein structures, and further studied their transcriptional responses to biotic (*Verticillium dahliae*, *Meloidogyne javanica*, *Cucumber Mosaic Virus*, and *Potato Virus Y* infections) and abiotic (cold stress) stimuli. Tomato defensin sequences were classified into two groups (C8 and C12). Our data indicate that the transcription of defensin coding genes primarily depends on the specific pathogen recognition patterns of *V. dahliae* and *M. javanica*. The immunodetection of plant defensin 1 protein was achieved only in the roots of plants inoculated with *V. dahliae.* In contrast, the almost null effects of viral infections and cold stress, and the failure to substantially induce the gene transcription suggest that these factors are probably not primarily targeted by the tomato defensin network.

## 1. Introduction

Plants are exposed to a plethora of biotic and/or abiotic stresses during their life cycle. To increase the likelihood of survival and successful reproduction, they need to develop countermeasures. As a result, the evolutionary race against pathogens and the struggle to endure adverse environmental conditions have resulted in the development and establishment of several defense mechanisms against these threats [1,2]. Furthermore, punctual mobilization of a multi-level response can be critical for survival. Timely induced host resistance is especially important during the initial stages of pathogen attack, where infection can be successfully contained, and thus the threat can be eliminated or at least diminished [3,4,5].

Plant cells can recognize some conserved non-self-molecules, described as Microbe-Associated Molecular Patterns (MAMPs) [6]. These structures are chemically categorized as lipopolysaccharides, peptides (elongation factors, elicitins, and flagellin), peptidoglycans, or polysaccharides (chitin fragments) [7]. In addition, plants can identify Damage-Associated Molecular Patterns (DAMPs) that are host biomolecule derivatives, indicative of cellular damage [2]. DAMPs are usually cytosolic or nuclear proteins that are apoplastically exposed and denatured upon infection. Moreover, MAMPs and DAMPs may act as signals of danger (perceived via specific pattern recognition receptors localized on plant cellular membranes [8]), and in turn, promote an array of defensive mechanisms; including the induction of resistance responses and the biosynthesis of immune-related proteins. Other fundamental responses, such as hypersensitive reaction, result in cell wall rigidification, and the synthesis of defense-related metabolites [9].

Defensins are widely distributed across eukaryotes and form the only known common group of Anti-Microbial Peptides (AMPs) detected in both vertebrates and invertebrates, as well as in plants [10]. They are composed of a small number of residues (18–45) usually including up to eight cysteines. The majority of defensin act by binding to the pathogens’ cell membrane, and when embedded, they formulate porous membranes that allow efflux of vital nutrients and ions. Plant defensins are characterized as small, highly stable, cysteine-rich cationic peptides possessing a characteristic cysteine-stabilized alpha-beta protein fold [10]. Despite their structural similarities, plant defensins show great amino acid diversity, while minor structural modifications can dramatically modify activity spectra [11].

Spatially, the transcriptions of the defensin-encoding genes have been described as ubiquitous in all plant organs [12,13,14]. Furthermore, it has been demonstrated that apart from their key role against pathogens in planta, defensins also regulate other biological processes mainly associated with growth and development [15,16]. In several species, a body of evidence is accumulating regarding the mobilization of anti-fungal defensin mechanisms [17] and the effectiveness of defensins against several fungi, such as *Phytophthora infestans* [14], *Verticillium dahliae*, and *Neurospora crassa* [16]. Although evidence suggests that the defensin mode of action (MOA) is more diverse than sole disease resistance, their participation in physiological processes upon viral infections and nematode infestations has received less attention [18,19,20].

The current study aimed at examining both the structure (in silico) of defensins and their transcriptional regulation under several biotic (fungal, viral, and nematode infection) and abiotic (cold stress) stimuli, in tomato. Taken together, these results shed light on the elucidation of the potential implications of defensin genes against diverse and devastating diseases. The obtained data can also be useful as a guideline for future research towards the optimization of exploiting the plant innate immunity system against pathogens.

## 2. Results

### 2.1. Defensin Structure Analysis

In order to identify possible organelle targeting areas of the tomato defensin proteins, a prediction of subcellular location (Supporting information 1) and the extent of transmembrane regions (Supporting information 2) were calculated in silico. Based on probability estimations, it was determined that all studied peptides were referring to secretory proteins (Table 1). The theoretical isoelectric point of these proteins was also computed [21]. Interestingly, the majority of tomato defensin proteins seem to possess a positive charge at physical cellular conditions (Table 1). Nevertheless, the isoforms SlDEF2a, SlDEF2b, and SlDEF3 deviated from the general rule and were several fold less basic (their PI was adjacent to the neutral pH range).

**Figure 1:**
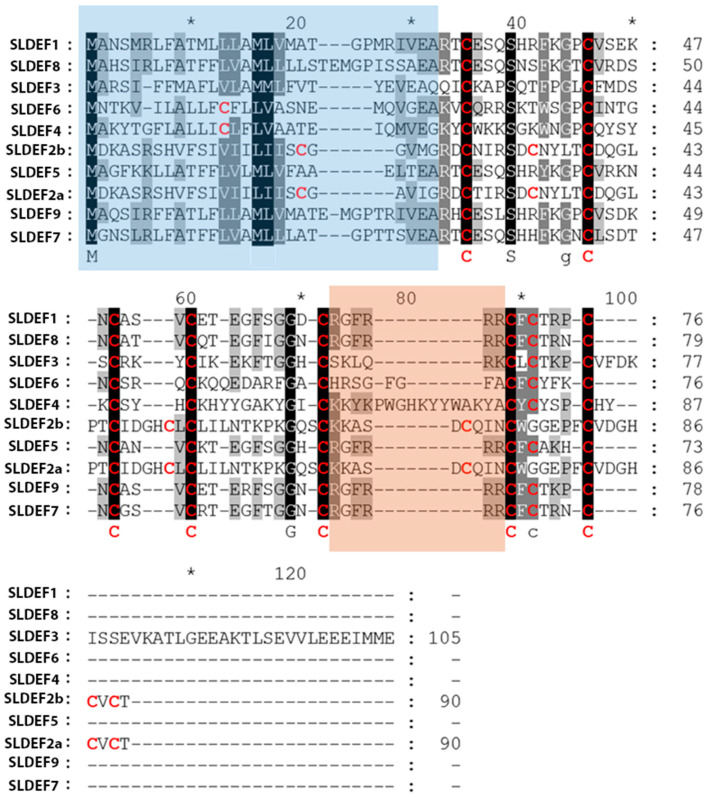
Residues’ alignment reveals a lack of amino acid sequence conservation in tomato plant defensins. Alignment of 10 tomato accessions. The conserved residues are in black-grey boxes, while cysteine residues are in red font. The blue box indicates the transit peptides, as predicted by the TargetP 1.1 Server (http://www.cbs.dtu.dk/services/TargetP/), and the orange box delimits the rich in basic amino acids loop. SlDef: *Solanum lycopersicum* defensin. * denotes odd number decades.

Moreover, based on the alignment (Figure 1), it was evident that the amino acid sequence variations were extreme in both the N-terminal transit peptide and the mature domain; hence revealing great heterogeneity. Nonetheless, we could conclude that a minimum of topology constraints (regarding the number/spacing of residues across cysteines) was retained. Most accessions possessed the motif C-X_10_-C-X_5_-C-X_3_-C-X_9_-C-X_6_-C-X_1_-C-X_3_-C and had eight cysteine residues in the mature structure. However, there were instances where minor alterations were observed. In the SlDEF4 and SlDEF6 accessions, more than six amino acids were detected among the fifth and sixth cysteine residues, thus resulting in an altered motif of C-X_10_-C-X_5_-C-X_3_-C-X_9_-C-X_16_-C-X_1_-C-X_3_-C and C-X_10_-C-X_5_-C-X_3_-C-X_9_-C-X_9_-C-X_1_-C-X_3_-C, respectively.

In order to achieve a prediction on the topology of disulfide bonds among cysteine residues (Table 1), an additional analysis using the DiANNA online server [22] was accomplished. The majority of peptides had the 1–8, 2–5, 3–6, 4–7 cysteine connectivity, as recently reported for C8 defensins [23]. Nevertheless, a few exceptions were detected: The C8 accession SlDEF4 had the 1-2, 3-6, 4-5, 7-8 configuration, and two tomato defensin isoforms possessed 12 cysteine residues.

In addition to the highly conserved cysteine numbers and spacing across all peptides, there were corresponding additional conserved residues detected at several amino acid positions, signifying a conserved functional inference. At position 39 (aligned proteins; Figure 1), a conserved serine residue was detected, while at position 69, a glycine residue was retained for all accessions (Figure 1). Furthermore, it was established that a significant percentage of the mature domain residues were of basic nature; predominantly arginine and lysine (Supporting information 3).

In order to estimate the phylogenetic relationships across accessions, a dendrogram was calculated (Supporting information 4) and two structural clusters were confirmed, also corresponding to peptides having eight and 12 cysteine residues, respectively. Further analyses were performed, explicitly for the mature domains alone, since defensin pro-domains possess no informative structure and can be lost or gained in several instances [23].

**Figure 2:**
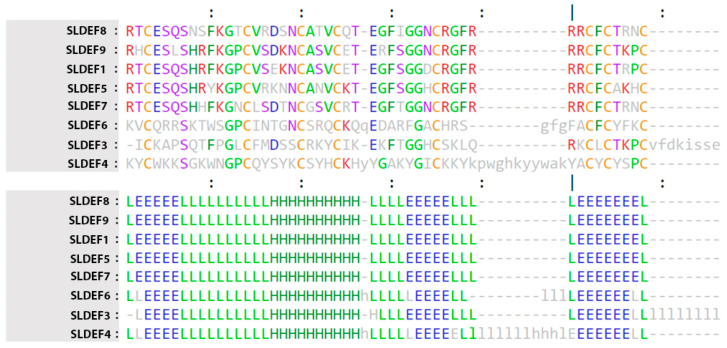
Each accession is shown in the pairwise Dali-alignment. Uppercase means structurally equivalent positions to SlDEF8. Lowercase indicates insertions relative to SlDEF8. The first part depicts the amino acid sequences of the selected neighbors. The second part shows the secondary structure assignments by DSSP (H/h: helix, E/e: strand, L/l: coil). The most frequent amino acid type is colored in each column. *SlDef*: *Solanum lycopersicum* defensin.

Additionally, structure comparison using the Dali server revealed a conserved tertiary structure for tomato defensin proteins despite the extended amino acid discrepancies. The three-dimensional structure of the model (Figure 2) identified the following two chief sections: The N-terminus consisted of a highly conserved beta-strand and α-helix structure, while the C-terminus (that was formed by more disordered structures) contained two beta-strands. Even though the C-terminal was less conserved than the N-terminal, the extra amino acids present in accession SlDEF4 did not significantly alter the strand-coil-strand domain. Furthermore, we generated a sequence profile around the structurally predicted proteins and the resulting stacked residues were depicted against the query protein, thus delimiting the residues having a functional significance across defensin types (Supporting information 5).

**Figure 3:**
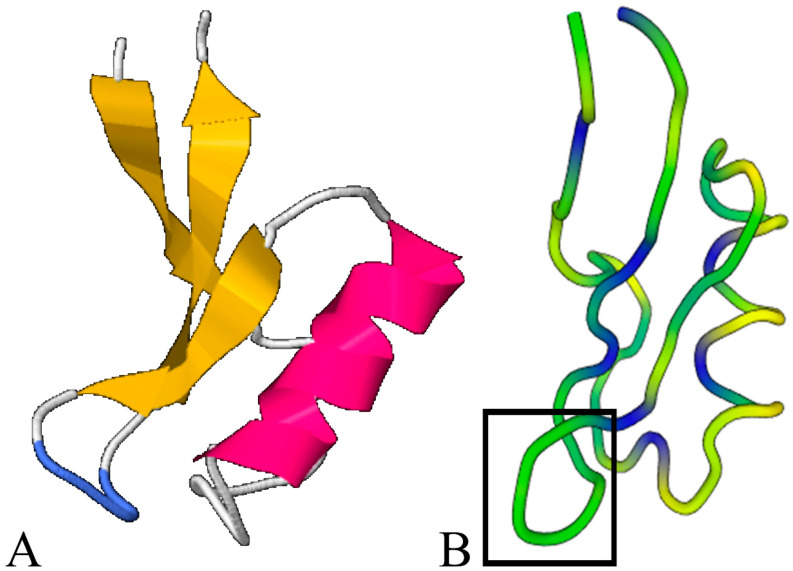
Structural similarity across tomato defensins. (**A**). Example of the conserved secondary structure (beta-strands, alpha-helix, and loops) as depicted for the SlDEF8 accession. (**B**). Sequence conservation across the superimposed tomato defensins (blue color denotes the conserved amino acids depicting the cysteine residues). The less conserved loop between the second and third beta-strand is also designated (black box).

Using homology modeling for the tomato defensins, 3D structure predictions were achieved (Table 2; Figure 3). Out of the ten different peptides, eight resulted in a database coverage of more than 85% and a confidence level that varied from 96% to 100%. The remaining two isoforms (snakins’ subtype) had low amino acid coverage (27–28%), despite the high confidence of the algorithm (circa 85%) and were removed from further analyses.

### 2.2. Defensin Responses Depend on Stimulus

**Figure 4:**
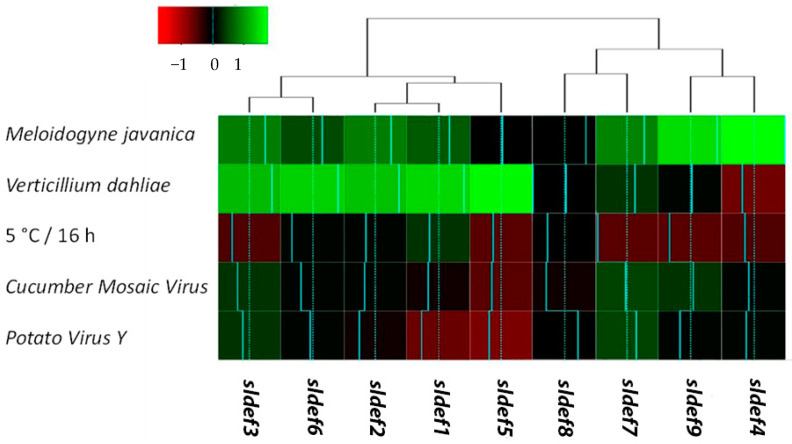
Heat map of relative transcription for tomato defensin genes exposed to discrete biotic/abiotic stresses. Relative mRNA abundance was assessed by real-time RT-qPCR, employing three independent biological replicates. Data were standardized across treatments. Up-regulation is delimited with green color; down-regulation is designated with red color. Two major hierarchical clusters according to stimulus–response are denoted. Statistical analysis for the treatments presented here is provided in the Supporting information 6 figure. *SlDef*: *Solanum lycopersicum* defensin.

In the current study, we aimed to describe the molecular crosstalk among common but discrete soil-borne pathogens of tomato (*Verticillium dahliae* and *Meloidogyne javanica*) and the plant immunity system; specifically, defensins. In addition, we investigated the possibility of host immunity responses to concurrent biotic/abiotic stress applied via common viruses (Cauliflower Mosaic Virus (CMV) and Potato Virus Y (PVY)) and cold treatment, respectively. In general, the tomato defensin genes’ transcriptional responses could be classified into two distinctive hierarchical groups according to pathogen type (Figure 4; Supporting information 6). 

Genes *sldef1*, *sldef2*, *sldef3*, *sldef5,* and *sldef6* presented a somewhat comparable transcription pattern, while isoforms *sldef4*, *sldef7*, *sldef8*, and *sldef9* were grouped distinctively from the first core of transcripts, forming a second hierarchical cluster. The defensins’ expression patterns seem neither to correspond to structural homologies nor to correlate to similar physicochemical properties of peptides. Nonetheless, it seems that there is a common pattern of recognition across specific defensin accessions, with a dependent differential response to stimuli (nematode versus fungal activation). More specifically, *Verticillium dahliae* infection resulted in a general up-regulation for the majority of defensin-related genes, while a significant upregulation of the *sldef4* and *sldef9* isoforms was noted as a response to *Meloidogyne javanica*. In addition, it seems that cold stress, as well as the viral infestation of tomato seedlings (via CMV and PVY infiltration), do not correspond to any noteworthy molecular responses. As a result, transcription rates across defensin accessions seem to be stable or somewhat downregulated. In order to detect whether the transcriptional response of defensins correlates to the proteomic level, a western blot analysis was performed across treatments using a defensin polyclonal antibody (Agrisera) with verified reactivity against Arabidopsis PDF1. Interestingly, across the diverse treatments, a band having the approximate molecular weight of 17 kD was vividly detected corresponding to a dimeric defensin protein, as a response to *Verticillium dahlia* infection in tomato roots.

## 3. Discussion

In the current study, the Sol Genomics Network (https://solgenomics.net/) and NCBI databases (https://www.ncbi.nlm.nih.gov/) were simultaneously screened in order to identify isoforms of tomato defensin genes for structural and transcriptional characterization, as a response to biotic/abiotic stress. Despite significant amino acid discrepancies across isoforms, it was established that the 3D structural conformation of tomato defensin proteins is rather conserved. Moreover, it seems that the tomato defensin gene members have a discrete transcriptional response, that is heavily stimulus-dependent (mostly nematode- or fungus-induced). The findings of this study are further discussed.

### 3.1. Structural Analysis

Two main peptide categories could be distinguished, according to the precursor defensin molecules [24]: Class I, including proteins with a signal peptide and a mature defensin domain, and Class II, delimiting polypeptides that have an additional acidic C-terminal pro-peptide (of approximately 33 residues). The latter is mainly required for targeting the vacuole and for peptide detoxification via transport across the plant secretory trail [25]. The majority of tomato defensin proteins of this study had a stop codon directly after the last cysteine residue (Figure 1), thus belonging to class I, while the SlDEF3 accession had a 32-residue glutamic acid-rich peptide (C-terminal).

The concurrent analysis of the cysteine residues’ number and position in the mature peptide core domain (Table 1) strengthens the view that the obtained tomato defensins can be generally classified into two groups: the cluster having C8 accessions and the one encompassing solely two peptides with 12 cysteine residues. The latter cluster seems to constitute a subfamily of antimicrobial defensins (snakins), originally described in potato [26]. These peptides fit a cluster of proteins encoded by snakin/GASA genes, which possess a Gibberellic Acid Stimulated Arabidopsis (GASA) domain of roughly 60 amino acids and 12 greatly preserved cysteine residues, which can form up to six disulfide bonds [27].

The majority of the tomato defensin peptides analyzed in the current study had the 1–8, 2–5, 3–6, 4–7 cysteine connectivity, as reported for C8 defensins. This is in accordance with several structural studies since plant defensins are characterized by two parallel disulfide bonds that connect the third beta-strand to the alpha-helix [23,28]. Moreover, it has been established that the evolution of disulfide bonds and tertiary structure undoubtedly occurs. For instance, C8 defensins have extensions (N- and C-termini) with an additional disulfide bridge compared to the C6 defensins [23]. Furthermore, the C10 petunia defensins substitute more than a few noncovalent interactions with additional disulfide bonds [29]. Greater deviations to disulfide connectivity, however, may not be well adapted [30].

The analyses across the different defensin proteins in tomato revealed that several conserved amino acids (predominantly serine and glycine) are retained at crucial positions in order to secure a conserved tertiary structure. The presence of hydrophobic as well as basic amino acids was also recorded. Such residues have been correlated to fungal lipid-binding capacity. The loop 5 domain of defensins can bind to lipids as proven with X-ray crystallography. These types (from the Solanaceae family) can also form dimers and have a high affinity for fungal sphingolipids [25]. Furthermore, higher antifungal activity has been detected towards mold fungi causing growth reduction and hyper-branching in hyphal tips, which has been attributed to the generally higher positive charge affected by the above-mentioned amino acid residues [31]. Finally, it has been reported [32] that other common residues include the two glycine residues (positions 12 and 32 relative to the plant defensin NaD1), an aromatic residue (at position 10), as well as a glutamate (at position 27), all of which participate in the folding of plant defensins [17].

### 3.2. Defensins Respond Differentially to Diverse Stimuli

Defensins are a multigene family detected in eukaryotes and are broadly distributed across plant families. Even though defensins are generally regarded as molecules with a primary role against pathogens [16], an increasing body of evidence supports a multifunctional role. Their implication as ion flux regulators [33], protein synthesis inhibitors, or cation tolerance mediators [34] has been established. Moreover, their heterologous expression in bacterial hosts [35] and defensin overexpression via genetic modification have shown that these peptides have a protective role against fungal colonization [14,36].

Particularly in tomato, Stotz and coworkers [15] highlighted the developmental role of a tomato defensin isoform for flower development and pollen viability. Furthermore, they demonstrated that overexpression of defensin proteins could provide significant foliar resistance against the *Botrytis cinerea* fungal pathogen. Recently, it was established that two defensin isoforms in tomato have a tissue-specific expression after *Phytophthora infestans* stress [14]. Even though transcript detection was attained across all tissues (root, stem, leaf, flower, and fruits), transcript levels greatly fluctuated. Nonetheless, it could be established that defensins were mainly detected in stems. Moreover, phytophthora infestation caused a statistically significant upregulation in leaves for the two genes studied (*sldef1* and *sldef9*).

In the current study, the transcription regulation of nine defensin genes was studied in root tissues of tomato seedlings after infection with the *Verticillium dahliae* fungal pathogen. According to the work of Cui and coworkers [14], we also established that several isoforms were significantly upregulated. Moreover, the transcript upregulation was confirmed with western immunoblotting, since a band at the approximate defensins’ molecular weight (circa 17KDa) was detected (data not shown). Arabidopsis *PDF1* appears as a multigene family, coding for highly similar peptides [37]. Arabidopsis PDF1 amino acid sequences are mostly similar to the peptide coded by the *sldef3* in tomato (Table 1) that was upregulated by the *V. dahliae* infection. This peptide has been characterized in tomato by Baxter and coworkers [38] as having antifungal properties after dimerization in order to mediate cell lysis. Thus, we postulate that the chemiluminescence signal corresponded to a dimeric antifungal defensin peptide, induced by *V. dahliae* infection. Nonetheless, discrepancies in transcriptional and proteomic detection can be acknowledged in the case of nematode infection. Antibody recognition can be extremely specific to an isoform epitope. Thus, transcriptional upregulation of a gene member (as a response to nematode infection) can be proven undetectable due to reduced reactivity of the antibody. Despite the fact that all tomato isoforms have a rather conserved conformation (conserved beta sheets and helixes), nevertheless, extreme amino acid differences can be identified at homologous positions (Figure 1), making protein detection a difficult task when using a heterologous antibody. Peptide oligomerization has also been observed in tobacco, a species closely related to tomato, a process that enhances the antifungal activity of the peptide [25,39]. Thus, our findings suggest that a wide-ranging initiation of immune-related responses was triggered by the fungal infection. It is well documented that fungal pathogens stimulate MAMPs-related immune responses via the activation of pattern recognition receptors they target [40]. Chitin, in particular, a major component of fungal cell walls, seems to be a key element for plant innate immune system recognition [41]. While a level of specificity in the MOA of plant defensins does exist, their activity mainly focuses on the disruption of fungal plasma membranes, which subsequently causes deregulation of the ionic balance, especially regarding the Ca^2+^, which is crucial for hyphal development. In some instances, peptides can even gain entrance into the fungal cells and directly cause cellular damage. Moreover, it has been reported that defensins also participate in the regulation of hypersensitive reaction, which is common during the development of fungal infection [35,42].

Plant defensins exhibit broad in vitro antifungal activity and present specific MOA against different genera of fungi. The stimuli for the induction of defensin production appear to be rather non-specific, as they can be produced by either pathogenic or non-pathogenic fungi [15,35,43]. Regarding the specific anti-fungal activities of plant defensins, a significant amount of data is available. Gaspar et al. [44] showed that transgenic cotton (expressing the defensin NaD1 from *Nicotiana alata*) achieved considerably higher levels of survival against *Fusarium oxysporum* f. sp. *Vasinfectum*, and *Verticillium dahliae*.

In the current study, we also studied the effect of *Meloidogyne javanica* presence on transcriptional regulation of defensins in tomato roots. In several cases, tomato defensins were positively regulated following nematode infection (Figure 4), which adds a novel perspective for plant defensins. Recently, it was established that elicitor peptides amplified immune responses of soybean against *Heterodera glycines* (soybean cyst nematode) and *Meloidogyne incognita* (a root-knot nematode) [45]. However, despite transcriptional upregulation, the PDF1 protein was not immunologically detected with the use of the PDF1 antibody. This could be the result of either discrepancy across the defensins’ epitopes or the relatively low levels of transcription of discrete defensin isoforms. Nonetheless, this finding indicates that the nematode and fungal stimuli differentially affect the regulation of the defensin system, while the underlying mechanisms that orchestrate the plant reactions against specific pathogens remain to be addressed.

Moreover, nematodes, unlike fungi, do not have chitin epidermis. Their cuticle consists mainly of collagen, which is covered by a carbohydrate-rich coating (surface coat), containing lipids and proteins [46]. The different composition of the nematode and fungi epidermis suggests that the recognition mechanisms of the innate immunity system must be unrelated. Indeed, differential activation is confirmed for different groups of defensin peptides [11]. These mechanisms, however, are more compound than simple membrane permeabilization prompted by several small antimicrobial peptides. Common characterized mechanisms include interfacing with explicit lipids, production of Reactive Oxygen Species (ROS), and stimulation of cell wall tension [17]. Still, nematodes employ complex strategies, such as delivering elicitors to suppress or bypass the plant defense mechanisms. In turn, plants sense the intracellular perturbations via the extracellular nucleotide-binding leucine-rich repeat (NR-LRR) immune receptors that recognize a wide range of effector proteins and PAMP-related patterns. Finally, reports also exist describing that plants recognize nematode pheromones and activate innate immune responses [47,48].

To test defensin responses to biotic and abiotic pressure in leaves, we applied cold stress and inoculation with two of the most severe viruses for tomato cultivation (CMV and PVY). Neither were specific transcription patterns observed across defensin genes, nor was the PDF1 protein detected. Moreover, across viral infections the patterns of defensin gene expression were almost comparable, signifying similar effects of both viruses. In contrast to fungal and nematode infections, it seems that viral infections of tomato leaves did not cause a systematic up-regulation of the defensin-related genes. On the contrary, for several isoforms it was established that a marginal relative decrease of transcripts can occur. This finding indicates that the effect of viral infection on the initiation of the defensin-related immune responses significantly differs from those of fungal and nematode infections. This result may be attributed to different contingencies. On one hand, the mechanism in the case of virus infection could be activated individually mostly by the DAMPs produced by cellular damage during the viral infection circle. This would be an indication of a discrete regulation correlating to MAMP-activated animal defensins [43]. On the other hand, the defensin system could be stimulated by viral MAMPs, but the level of mobilization could be significantly lower than that in fungal and nematode infections.

Nonetheless, it should be noted that Roberts et al. [49] recorded a systemic downregulation of a defensin gene (*PDF 2.1*) in Cauliflower mosaic virus-infected Arabidopsis plants. In addition, it has been reported that the plant innate immune trail is triggered by MAMPs stimulated specific Pattern Recognition Receptors [50,51]. This orchestrates metabolic adjustments including RNA silencing, ribosomal inactivation, salicylic acid-dependent responses, ROS signaling, and mobilization of the antioxidant mechanism. The aforementioned responses constitute the “first line of defense” against plant viruses and are often manifested as a hypersensitive reaction, which may lead to a plant-wide systemic acquired resistance (SAR) [51,52,53]. It appears that unlike animal defensins that target viral envelopes, glycoproteins, and capsids [43], plant defensins appear to have limited participation in the innate immune responses during viral infections.

Finally, we demonstrated that cold stress mostly downregulated the transcription of most of the genes coding for tomato defensin peptides. This finding contradicts observations of other research teams that recorded defensin induction in response to abiotic stress scenarios, including cold stress [24,54]. Available data suggest that some plant defensins hold specific developmental roles in some taxa (allowing them to withstand marginal environmental conditions), such as cold acclimation in winter wheat [54]. In contrast, other isoforms may be non-specifically stimulated by DAMPs produced by the disruption of the cellular membrane under severe abiotic stress. We cannot, however, uncritically rule out the possibility that under abiotic stress other specific physiological/metabolic procedures are induced at the expense of a more generalized defensin response.

Notwithstanding significant advances in elucidating the molecular mechanisms of plant-pathogen interplay and the significance of nonpathogenic microorganisms in induced resistance, the particulars of the procedures involved, principally the role of antimicrobial peptides, remain essentially undefined [55]. Hence, studies deciphering the induced resistance mechanisms are of pivotal significance for explaining plant immunity. Moreover, they have potential practical applications for the advancement of innovative disease control actions based on the stimulation of the plant defense mechanisms by microbial agents. Hence, in order to fully appreciate the palette of multifunctional roles of the defensin multigene family, further experimentation is needed.

## 4. Materials and Methods

### 4.1. Plant Material and Growing Conditions

For all experiments, a tomato variety (cv San Marzano nano- Vilmorin, La Ménitré, France) without any known resistance to viral, fungal, and nematode infections was used. The plants were grown in 0.5 L pots containing Compo Sana fertilizer-enriched substrate mix (Compo, Münster, Germany). In order to study the effects of viral, fungal, and nematode infection, as well as cold stress on defensins’ transcription, 10 tomato seedlings per treatment at the 4th to 5th true-leaf-stage (24 days post-transplantation) were artificially inoculated with the pathogens (for specific pathogen inoculation procedure see below). After infection, plants were grown for another 21 days in a growth chamber under a 12 h light/dark cycle and at 25 ± 1 °C air temperature. Tissues from leaf blades were sampled to test defensins’ regulation under viral infection and cold stress, while specimens from root tissues were selected in the case of *M. javanica* and *V. dahliae* infections (non-destructive sampling). For both sample types, an uninfected control tissue was also employed. For cold stress treatment, untreated seedlings 45 d after transplantation were placed in a chamber at 5 °C air temperature in the dark for 16 h. The collected samples from all treatments were immediately frozen in liquid nitrogen, homogenized using sterile pestles/mortars, and stored at −80 °C. All assays were conducted in triplicate.

### 4.2. Mechanical Inoculations with CMV and PVY Viruses

Leaf samples from a PVY (belonging to the necrotic group N) -infected *Solanum tuberosum* L. and a CMV-infected *Nicotiana benthamiana* Domin plant were used as live virus reference strains. The PVY presence on infected potato plants was verified by reverse-transcription polymerase chain reaction (RT-PCR) [56] and after mechanical inoculation onto *Nicotiana tabacum* L. cv. “Samsun” plants, severe veined necrosis was induced. The PVY-infected potato plants were also tested for the presence of Tomato spotted wilt virus (TSWV) [57], Potato virus *X* (PVX detection kits; Boehringer), CMV [58], and viruses belonging to the genus *Tobamovirus* [59], and none of the above-mentioned viruses were detected. The CMV-infected *N. benthamiana* was also tested for the presence of TSWV, and the virus was not detected.

For the preparation of the inoculum, leaf tissues were squashed in phosphate buffer (pH 7.0), and then mechanically inoculated onto carborundum-dusted leaves of healthy tomato plants, at the appropriate stage of development. For negative controls, carborundum-dusted/phosphate buffer-treated leaves were used. The successful infection was verified by RT-PCR by using appropriate primers for each plant individually [56,58].

### 4.3. Nematode Inoculation

**Figure 5:**
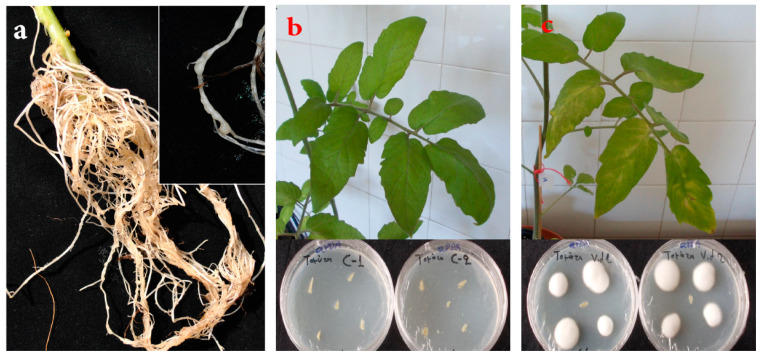
Visible knots on *M. javanica*-infected roots (**a**), and symptoms of *V. dahliae* infections in roots of control (**b**) and infected (**c**) tomato plants. In (**b**,**c**), bottom panels show Petri dishes from pathogen re-isolation.

Tomato seedlings were inoculated with a population of *Meloidogyne javanica* maintained in potted tomatoes at the Laboratory of Nematology of the Institute of Olive Τree, Subtropical Crops, and Viticulture (Heraklion, Crete, Greece). In brief, eggs were extracted from infected roots with sodium hypochlorite and incubated in an extraction dish at 25 °C [60]. Juveniles (J_2s_) hatched within 24 h were discarded. Nematodes collected after 72 h were used for plant infection at a rate of 100 J_2s_ per plant. After the cultivation period, as stated before, plants were uprooted and the remaining soil was carefully removed under running tap water. The root galling index (RGI) was assessed [61]. All inoculated plants exhibited visible nematode nodes on their secondary roots (Figure 5a) with a quite low RGI value ranging from 1 to 2.

### 4.4. Verticillium Dahliae Preparation and Plant Inoculation

The highly virulent *V. dahliae* isolate 998-1 originated from symptomatic eggplant (*Solanum melongena* L.) with proven pathogenicity on tomato was used in tomato *V. dahliae* bioassays [62]. *Verticillium dahliae* conidia were produced by growing the fungus in Potato Dextrose Broth (PDB) (Sigma-Aldrich, St Louis, Missouri, MO, USA) at 160 rpm and 24 °C for 5 days, harvested by filtration through four layers of cheesecloth, and the suspension was centrifuged at 3000× *g* for 10 min. Spores were re-suspended in sterilized distilled water and their concentration was adjusted to 1 × 10^6^ conidia/mL. Tomato seedlings were inoculated at the 4th to 5th true-leaf-stage. Plants were artificially inoculated with the fungus by root drenching (20 mL of conidial suspension). Control plants were mock-inoculated by root-drenching them with an equal amount of sterilized distilled water.

### 4.5. Verticillium Wilt Disease Assessment and Pathogen Re-Isolation

Visible symptoms of Verticillium wilt on tomato plants were recorded 20 and 30 days post-inoculation (dpi). The disease severity at each observation was calculated as a ratio of symptomatic leaves (exhibiting wilting, chlorosis, yellowing, and necrosis), compared to the total leaf number for each plant. At 30 dpi, plants were cut above the soil level, their leaves were removed, and longitudinal and transverse sections of stems were performed to observe vascular tissue discoloration. To verify the presence of *V. dahliae* in vascular tissues of tomato stems (inoculated and mock-inoculated seedlings), plants were surface-disinfected by spraying with 95% ethyl alcohol (Sigma-Aldrich, St Louis, Missouri, MO, USA) and quickly passed through a flame thrice. For each plant, five xylem chips (taken from different positions along the stem) were placed onto acidified potato dextrose agar (PDA) after the removal of the phloem. Plates were incubated at 22 °C in the dark for 10 days. The emerging fungi were examined under a light microscope and identified as *V. dahliae* according to their morphological characteristics [63] (Figure 5b,c, bottom).

### 4.6. In Silico Analyses

To identify defensin-encoded genes in the tomato genome (Table 1), a concurrent analysis in two different databases was conducted. The current tomato proteome in Sol Genomics Network (https://solgenomics.net/) was screened against previously characterized tomato defensin genes [64], using an E value threshold of e^−25^ and a query coverage larger than 80%. In addition, a BlastP query in the NCBI database (https://www.ncbi.nlm.nih.gov/) was conducted to identify genes coding for defensin proteins. Finally, proteins annotated as defensin/defensin-like sequences were also collected from Genbank. Redundant entries were removed and predictions of signal peptides and subcellular location of the deduced peptides were conducted using the TargetP 1.1 Server (http://www.cbs.dtu.dk/services/TargetP/) and the DeepLoc-1.0: Eukaryotic protein subcellular localization predictor (http://www.cbs.dtu.dk/services/DeepLoc/). Protein transmembrane regions were computed using the algorithm of the TMHMM Server v. 2.0 (http://www.cbs.dtu.dk/services/TMHMM/), while the ProtParam tool (https://web.expasy.org/protparam/) was employed to calculate the physical and chemical parameters of the proteins. The PI of defensins was also determined using the expasy server (https://web.expasy.org/compute_pi/). Finally, the Protein Structure Prediction Server (http://ps2.life.nctu.edu.tw/) was employed in order to detect homologous proteins and verify the tomato defensin protein structures. Proteins with significant structural similarity to characterized defensins were further processed and a prediction of each tomato protein domain structure was conducted using the Phyre2 web portal for protein modeling [65]. The prediction of disulfide bonds was performed by using the DiANNA 1.1 (http://clavius.bc.edu/~clotelab/DiANNA/), as well as the Disulfind (http://disulfind.dsi.unifi.it) online servers. Structural alignment of the resulting tomato defensin models and 3D superimposition were computed using the DALI web-server [66]. Amino acid multiple alignments were computed with the ClustalX program [67] under default parameters, while the alignment was depicted with genedoc [68]. Phylogenetic inferences were made by the implementation of MUSCLE alignment, Gblocks curation, and the PhyML algorithm [69].

### 4.7. RNA Extraction, Reverse Transcription, qPCR Amplification

Total RNA was extracted using a method previously described [70]. Briefly, plant tissue was grounded in lysis buffer 1/10 (*w*/*v*) (8 M GuHCl, 25 mM EDTA, 1% Sarcosyl, 2% Triton X-100, 25 mM sodium citrate, 0.2 M sodium acetate, and pH adjusted to 5.2 with acetic acid) (all chemicals were acquired from AppliChem GmbH, Germany). The lysate was incubated at 65 °C for 10 min and then centrifuged at 16,000× *g* for 10 min. Then, 500 μL of the supernatant was transferred to a fresh tube and 625 μL of absolute ethanol was added (to obtain 55.5% final concentration). Nucleic acids were bound to silica columns (FT-2.0 Filter-Tube Spin-Column System, G. Kisker GbR, Steinfurt, Germany) by centrifugation at 1500× *g* for 10 min. The column was washed once with 700 μL ‘wash buffer 1’ (4 M GuHCl, 25 mM Tris–HCl pH 6.6, and 60% ethanol) and twice (700 μL and 400 μL respectively) with ‘wash buffer2’ (2 mM Tris–HCl pH 7.0, 20 mM NaCl, and 80% ethanol) by centrifugation at 8000× *g* for 1 min. Nucleic acids were eluted using 70 μL of preheated (80 °C) nuclease-free elution buffer (10 mM Tris–HCl, pH 8.0). Finally, DNA was enzymatically removed with DNAse I (ThermoFisher Waltham, Massachusetts, USA). The absence of total DNA was verified with qPCR using primers for *UBQ* before reverse transcription (Supporting information 7).

cDNA synthesis was performed using the Superscript II cDNA synthesis kit (ThermoFisher, Waltham, USA) according to the manufacturer’s instructions. qPCR amplification was monitored via the PowerUp™ SYBR^®^ Green Master Mix (ThermoFisher, Waltham, MA, USA) using a QuantStudio 3 Real-Time PCR System (ThermoFisher, Waltham, MA, USA). For all samples, qPCR reactions were performed in triplicate. A pairwise fixed reallocation randomization test was performed via the REST-xl package, as reported by [71]. The UBQ gene was used as a reference gene and the untreated control for each treatment was used for calibration. The ∆∆CP method was used in order to calculate the relative transcription of genes across treatments [72]. For the heat map construction, gene profiles were processed: expression values were standardized (median-centered across each gene) and subjected to hierarchical clustering employing gplots version 3.0.1 (heatmap.2 command) in R.

### 4.8. Statistical Design and Analysis

Statistics for relative expression was performed using Statgraphics Centurion (Statpoint Technologies, Warrenton, VA, USA). Significant differences between treatments were determined by one-way ANOVA by least significant difference (*p* < 0.05).

## 5. Conclusions

Mining pipelines for tomato defensins were proven to be greatly effective. Under strict criteria, ten protein accessions were identified. According to the cysteine residues’ number and resulting motifs, tomato defensins were classified into two groups (C8 and C12). Within these clusters, subcategories of comparable sequences could be distinguished. Structural elucidation confirmed a conserved mature domain consisting of one alpha-helix and three beta-strands, while conserved cysteine, glycine, and arginine residues were detected across accessions. Prediction of disulfide bonds revealed that sub-structural diversity might exist. However, the structural variety of tomato defensins did not correlate to the transcriptional differential regulation and their role in plant defense. Pattern transcriptional analysis for the defensin-related genes under the influence of several biotic factors and cold stress revealed interesting conclusions that contribute to the elucidation of plant innate immunity mechanisms. The transcription of most defensin-related genes was significantly up-regulated under the influence of fungal and nematode infections. The PDF1 protein, however, was detected only after the fungal infection. In contrast, viral infections caused a non-conclusive effect, while cold stress caused a slight downregulation of gene transcription. In both cases, no detection of PDF1 protein was achieved. These results suggest that the initiation of the defensin system depends mostly on specific pattern recognition of pathogens. *Verticillium dahliae* and *Meloidogyne javanica* were able to substantially stimulate the transcription of most defensin genes. However, such stimulation was not observed for the CMV and PVY viruses, suggesting that the defensin system lacks specificity for viruses in tomato.

## Figures and Tables

**Table 1:** Defensin accession numbers, protein secretion probability, and cysteine motifs/connectivity.

a/a	Acronym	NCBI Accession (Nucleotide/Protein)	SOLYC Number	Motif	Theoretical PI	Cys	Predicted Connectivity	Secretion Probability
1	SlDEF1	NM_001247943/NP_001234872	Solyc07g007750.3.1	C-X_10_-C-X_5_-C-X_3_-C-X_9_-C-X_6_-C-X_1_-C-X_3_-C	8.96	8	1–8, 2–5, 3–6, 4–7	0.934
2	SlDEF2a	XR_003246738/XP_025886757	Solyc05g150107.1.1	C-X_5_-C-X_4_-C-X_6_-C-X_4_-C-X_1_-C-X_11_-C-X_5_-C- C-X_3_-C-X_6_-C-X_4_-C-X_1_-C	6.76	12	1–10, 2–5, 3–8, 4–7, 6–9	0.981
3	SlDEF2b	XR_003246738/XP_019069532	Solyc05g150107.1.1	C-X_5_-C-X_4_-C-X_6_-C-X_4_-C-X_1_-C-X_11_-C-X_5_-C- C-X_3_-C-X_6_-C-X_4_-C-X_1_-C	6.76	12	1–10, 2–5, 3–8, 4–7, 6–9	0.957
4	SlDEF3	NM_001328663/NP_001315592	Solyc07g006380.3.1	C-X_10_-C-X_5_-C-X_3_-C-X_9_-C-X_6_-C-X_1_-C-X_3_-C	6.56	8	1–8, 2–5, 3–6, 4–7	0.989
5	SlDEF4	XM_004242790/XP_004242838	Solyc07g009060.4.1	C-X_10_-C-X_5_-C-X_3_-C-X_9_-C-X_16_-C-X_1_-C-X_3_-C	9.25	8	1–2, 3–6, 4–5, 7–8	0.969
6	SlDEF5	XM_010321070/XP_010319372	Solyc04g008470.3.1	C-X_10_-C-X_5_-C-X_3_-C-X_9_-C-X_6_-C-X_1_-C-X_3_-C	9.54	8	1–8, 2–5, 3–6, 4–7	0.984
7	SlDEF6	XM_004242803/XP_004242851	Solyc07g009260.3.1	C-X_10_-C-X_5_-C-X_3_-C-X_9_-C-X_9_-C-X_1_-C-X_3_-C	8.99	8	1–8, 2–5, 3–6, 4–7	0.883
8	SlDEF7	NM_001310317/NP_001297246	Solyc07g007710.4.1	C-X_10_-C-X_5_-C-X_3_-C-X_9_-C-X_6_-C-X_1_-C-X_3_-C	8.98	8	1–8, 2–5, 3–6, 4–7	0.954
9	SlDEF8	NM_001310318/NP_001297247	Solyc07g007730.4.1	C-X_10_-C-X_5_-C-X_3_-C-X_9_-C-X_6_-C-X_1_-C-X_3_-C	8.77	8	1–8, 2–5, 3–6, 4–7	0.980
10	SlDEF9	NM_001346524/NP_001333453	Solyc07g007755.1.1	C-X_10_-C-X_5_-C-X_3_-C-X_9_-C-X_6_-C-X_1_-C-X_3_-C	9.14	8	1–8, 2–5, 3–6, 4–7	0.951

Secretion probability as predicted by the TargetP 1.1 server (http://www.cbs.dtu.dk/services/TargetP/).

**Table 2:** 3D structure prediction using the Phyre2 homology protein modeling.

a/a	Code	NCBI Accession (P)	SOLYC Number	Confidence	Coverage	Template
1	SlDEF1	NP_001234872	Solyc07g007750.3.1	100%	100%	c2lr3A ^1^
2	SlDEF2a	XP_025886757	Solyc05g150107.1.1	86.3%	28%	d1f2si ^2^
3	SlDEF2b	XP_019069532	Solyc05g150107.1.1	85.9%	27%	c1f2sI ^2^
4	SlDEF3	NP_001315592	Solyc07g006380.3.1	99.9%	100%	c4uj0B ^1^
5	SlDEF4	XP_004242838	Solyc07g009060.4.1	96.2%	83%	c2n2qA ^1^
6	SlDEF5	XP_010319372	Solyc04g008470.3.1	99.9%	100%	c2lr3A ^1^
7	SlDEF6	XP_004242851	Solyc07g009260.3.1	100%	98%	d1bk8a ^1^
8	SlDEF7	NP_001297246	Solyc07g007710.4.1	99.9%	100%	c2lr3A ^1^
9	SlDEF8	NP_001297247	Solyc07g007730.4.1	100%	100%	c2lr3A ^1^
10	SlDEF9	NP_001333453	Solyc07g007755.1.1	100%	100%	c2lr3A ^1^

^1^ Defensins/Defensins like proteins, ^2^ snakins like proteins deposited in Protein Data Bank (PDB; https://www.rcsb.org/). SlDef: *Solanum lycopersicum* defensin.

## Supplementary Materials

Supplementary materials can be found at https://www.mdpi.com/1422-0067/21/24/9380/s1; Supporting information 1:.DeepLoc 1.0 localization prediction for *Solanum lycopersicum* defensin genes; Supporting information 2: Signal peptide prediction for *Solanum lycopersicum* defensin genes; Supporting information 3: Proportion of Amino acids across *Solanum lycopersicum* defensin genes; Supporting information 4: Phylogenetic relationships across defensin genes; Supporting information 5: Stacked sequence profiles of tomato defensin accessions; Supporting information 6: Relative transcription for tomato defensin genes exposed to discrete biotic/abiotic stresses. Relative mRNA abundance was assessed by real-time RT-qPCR. Data were standardized as reported in the Material and Methods segment. Significant differences between treatments were determined by ANOVA and post-hoc comparisons by least significant difference (*p* < 0.05). Bars represent means (±SE) of three biological replications. Vertical axis shows the relative expression to the control gene Ubiquitin; Supporting information 7: Primers used for the amplification of *Solanum lycopersicum* defensin genes (*S1Def1*–*S1Def9*) across treatments, and *Ubiquitin* (*UBQ*) control.

## Abbreviations

CMV	Cucumber Mosaic Virus
DAMP	Damage-Associated Molecular Patterns
MAMPs	Microbe-Associated Molecular Patterns
PVY	Potato Virus Y
