# Supplementary material for: Structural Diversity and Highly Specific Host-Pathogen Transcriptional Regulation of Defensin Genes Is Revealed in Tomato
Source: Int J Mol Sci. 2020 Dec 9;21(24):9380. doi: 10.3390/ijms21249380 (PMC7764197; doi:10.3390/ijms21249380)

Int. J. Mol. Sci. 2020, 21, x; doi: FOR PEER REVIEW

Nikoloudakis et al.

Structural diversity and highly specific host-pathogen transcriptional regulation of defensin genes is revealed in tomato seedlings

Supporting information 1: DeepLoc-1.0 localization prediction for *Solanum lycopersicum* defensin genes

NP_001315592.1
Prediction: Extracellular, Soluble

Localization	Extracellular	Lysosome/Vacuole	Endoplasmic reticulum	Cell membrane	Golgi apparatus	Cytoplasm	Mitochondrion	Plastid	Nucleus	Peroxisome
**Likelihood**	0.9762	0.0156	0.0062	0.0019	0.0001	0	0	0	0	0

Type	Soluble	Membrane
**Likelihood**	1	0

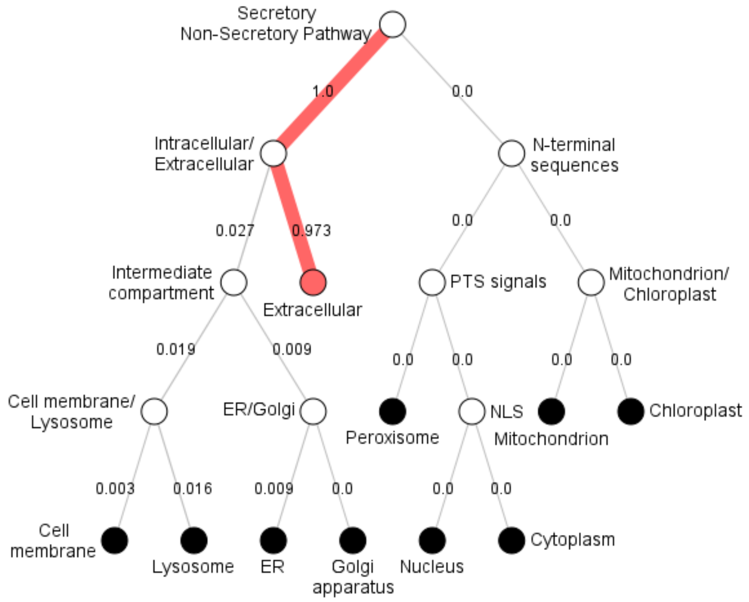

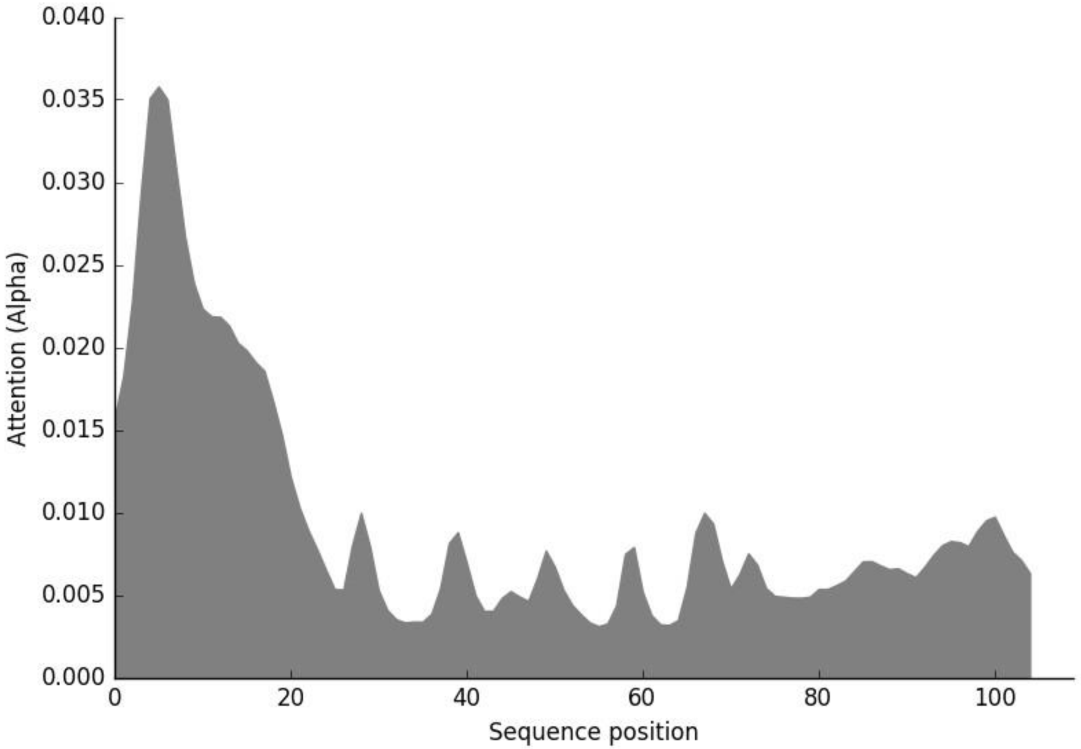

XP_004242851.1
Prediction: Extracellular, Soluble

Localization	Extracellular	Lysosome/Vacuole	Endoplasmic reticulum	Cell membrane	Cytoplasm	Golgi apparatus	Mitochondrion	Plastid	Nucleus	Peroxisome
**Likelihood**	1	0	0	0	0	0	0	0	0	0

Type	Soluble	Membrane
**Likelihood**	1	0

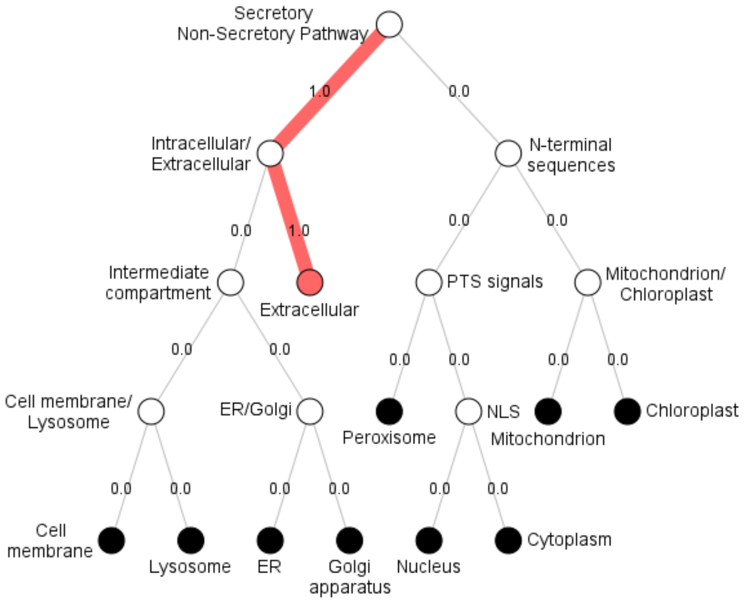

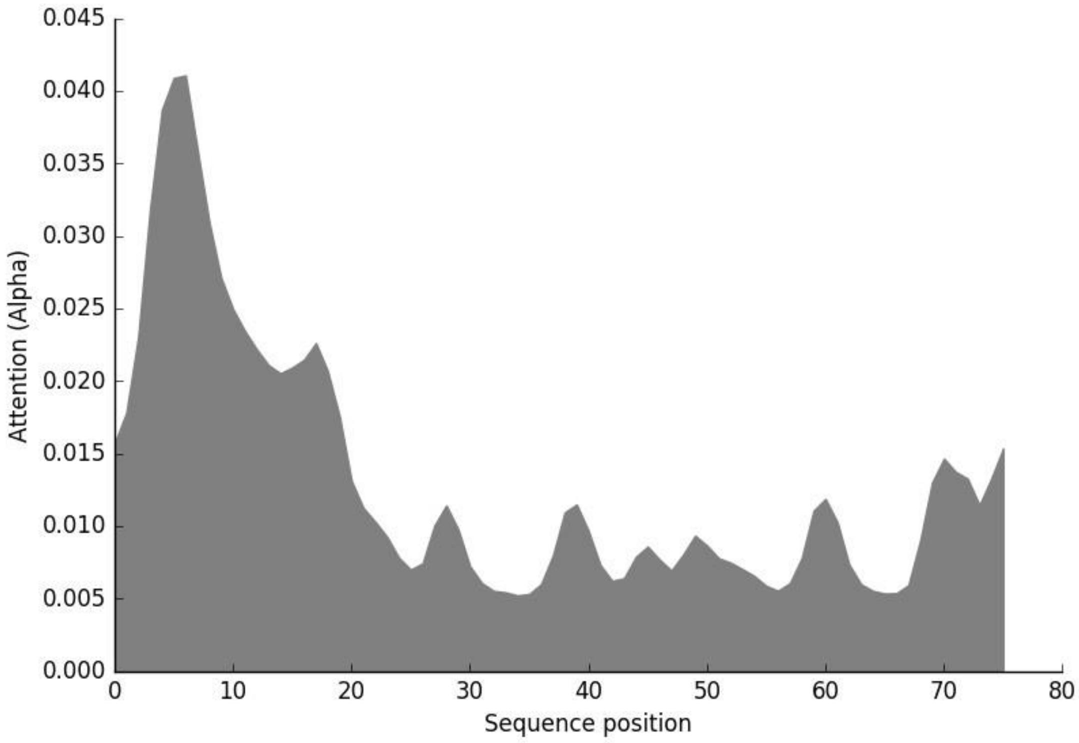

XP_010319372.1

Prediction: Extracellular, Soluble

Localization	Extracellular	Lysosome/Vacuole	Endoplasmic reticulum	Cell membrane	Golgi apparatus	Mitochondrion	Cytoplasm	Plastid	Nucleus	Peroxisome
**Likelihood**	1	0	0	0	0	0	0	0	0	0

Type	Soluble	Membrane
**Likelihood**	1	0

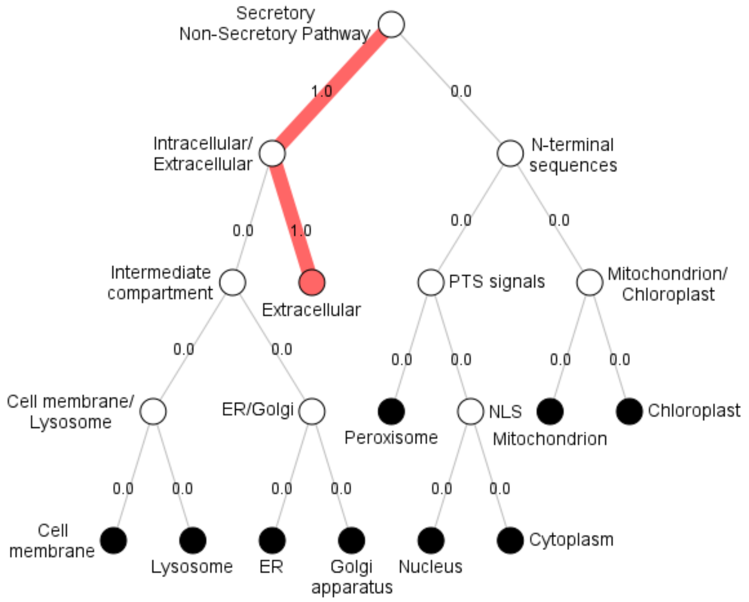

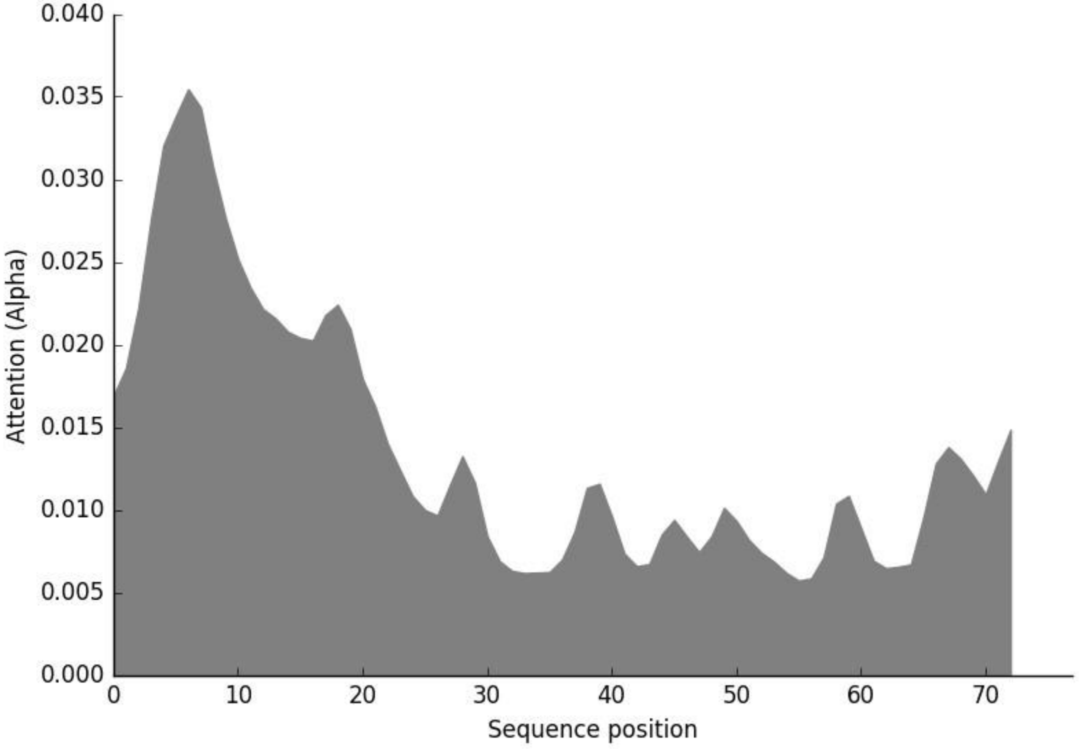

XP_025886757.1
Prediction: Extracellular, Soluble

Localization	Extracellular	Cell membrane	Lysosome/Vacuole	Endoplasmic reticulum	Golgi apparatus	Cytoplasm	Mitochondrion	Nucleus	Plastid	Peroxisome
**Likelihood**	0.9996	0.0003	0.0001	0	0	0	0	0	0	0

Type	Soluble	Membrane
**Likelihood**	0.9982	0.0018

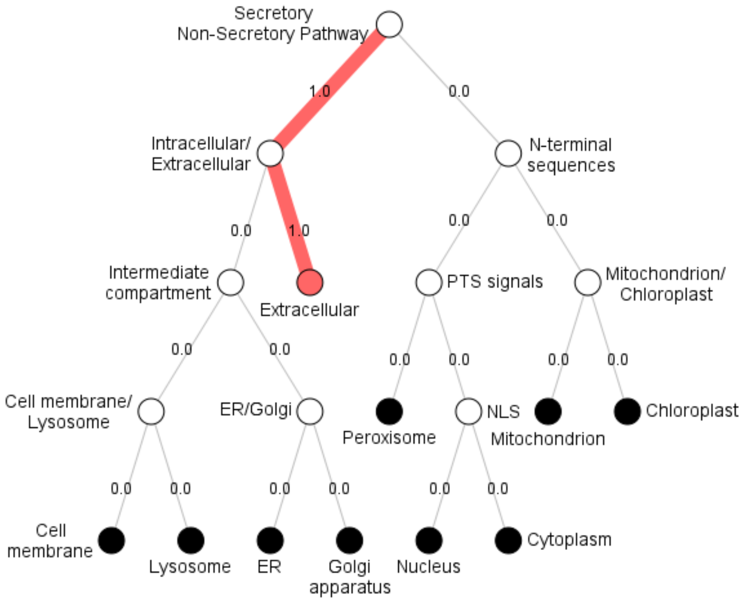

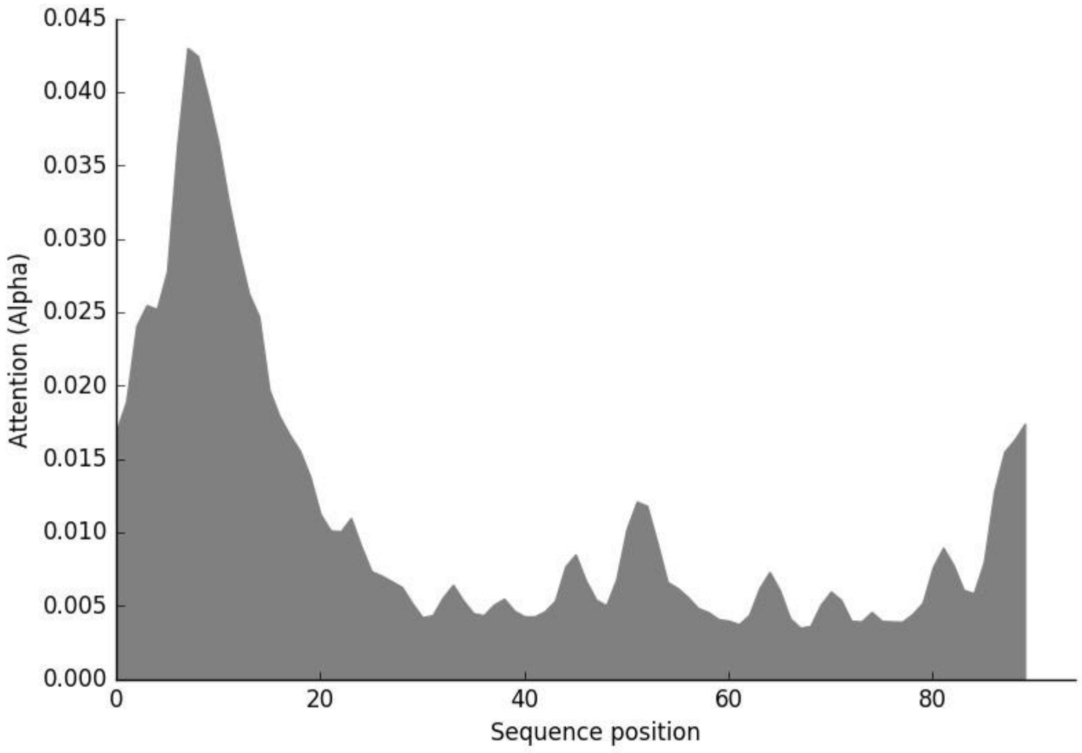

NP_001333453.1

Prediction: Extracellular, Soluble

**Localization**	**Extracellular**	**Lysosome/Vacuole**	**Endoplasmic reticulum**	**Cell membrane**	**Cytoplasm**	**Golgi apparatus**	**Mitochondrion**	**Plastid**	**Nucleus**	**Peroxisome**
**Likelihood**	0.9999	0	0	0	0	0	0	0	0	0

**Type**	**Soluble**	**Membrane**
**Likelihood**	1	0

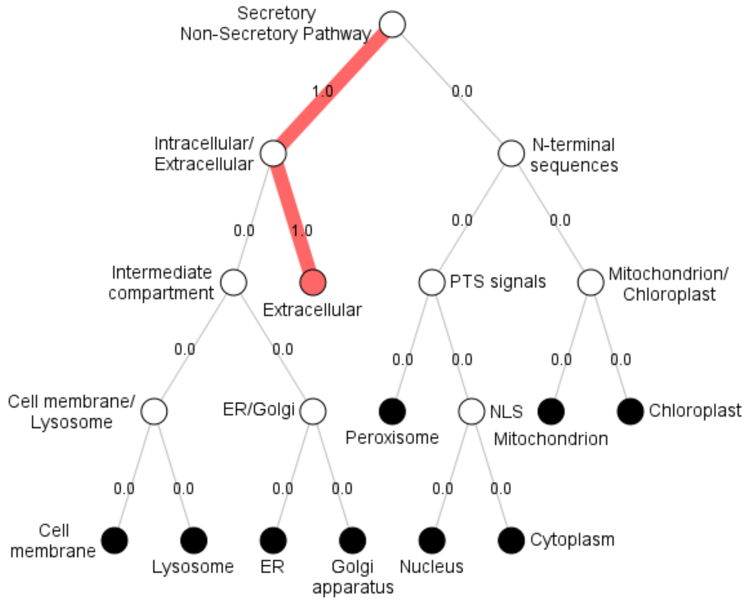

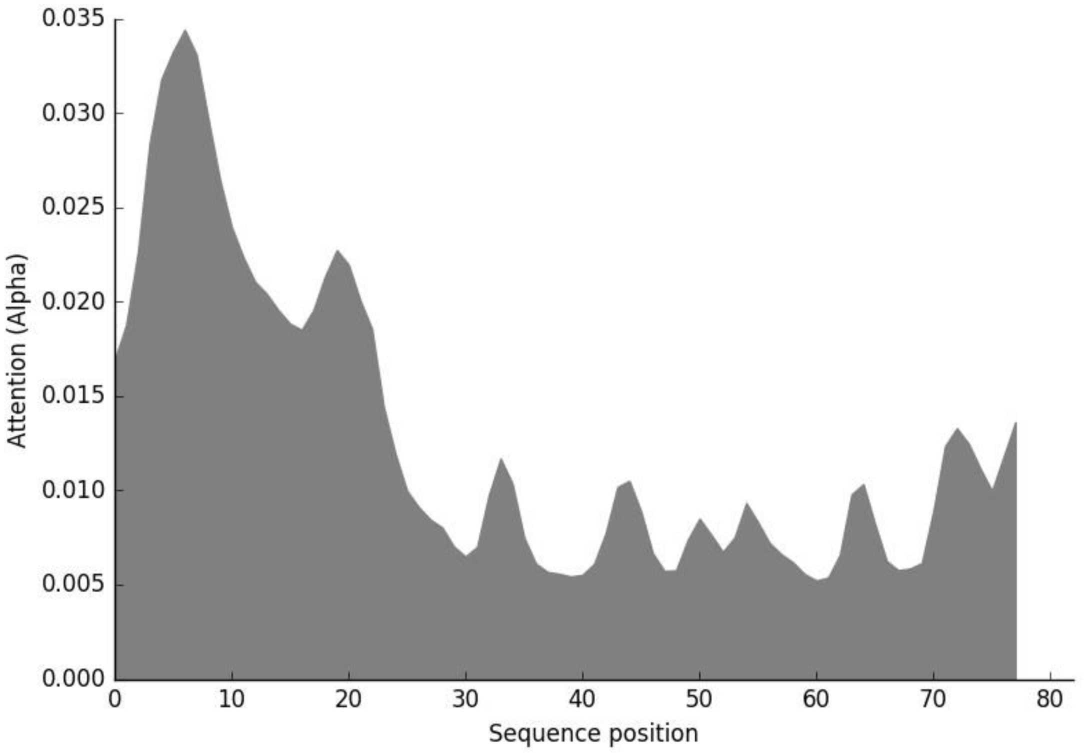

XP_004242838.1

Prediction: Extracellular, Soluble

Localization	Extracellular	Lysosome/Vacuole	Endoplasmic reticulum	Cytoplasm	Cell membrane	Golgi apparatus	Mitochondrion	Plastid	Nucleus	Peroxisome
**Likelihood**	1	0	0	0	0	0	0	0	0	0

Type	Soluble	Membrane
**Likelihood**	1	0

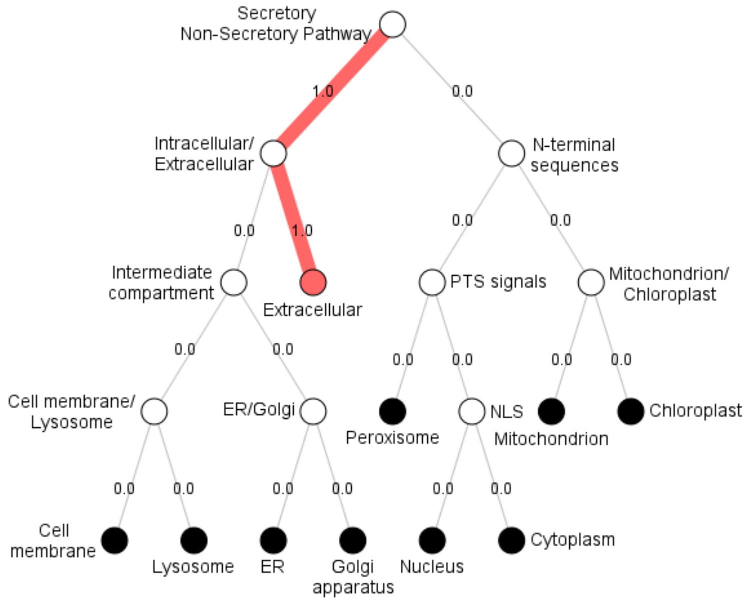

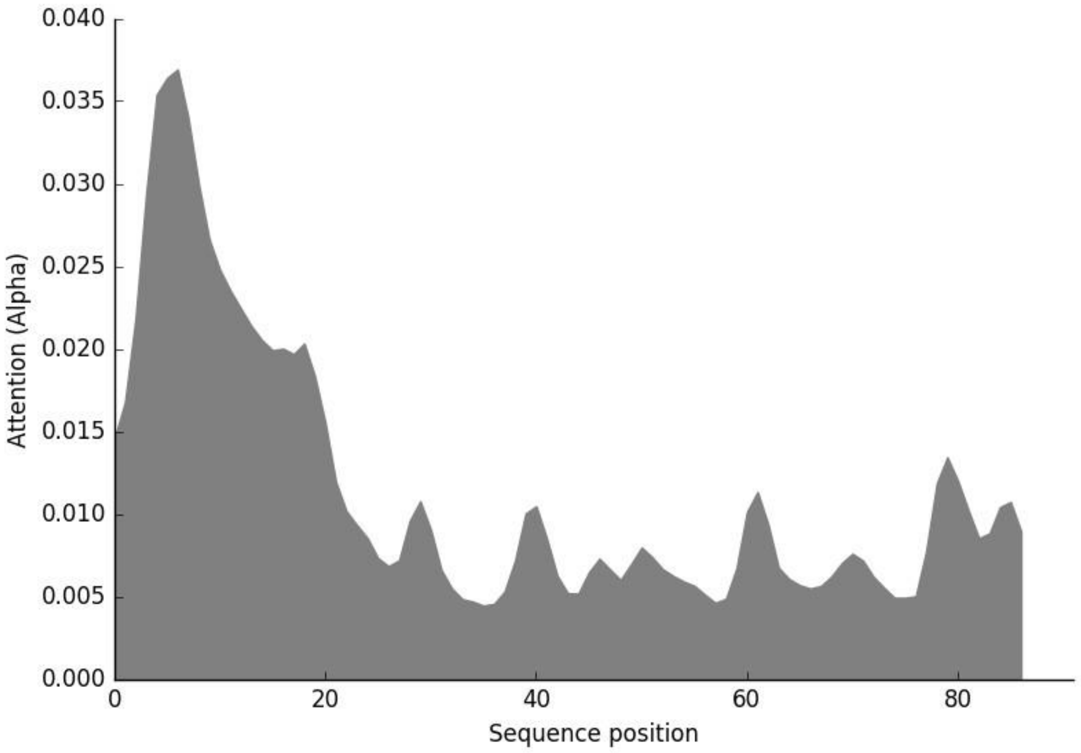

NP_001297246.1

Prediction: Extracellular, Soluble

Localization	Extracellular	Lysosome/Vacuole	Endoplasmic reticulum	Cell membrane	Mitochondrion	Cytoplasm	Golgi apparatus	Plastid	Nucleus	Peroxisome
Likelihood	1	0	0	0	0	0	0	0	0	0

Type	Soluble	Membrane
Likelihood	1	0

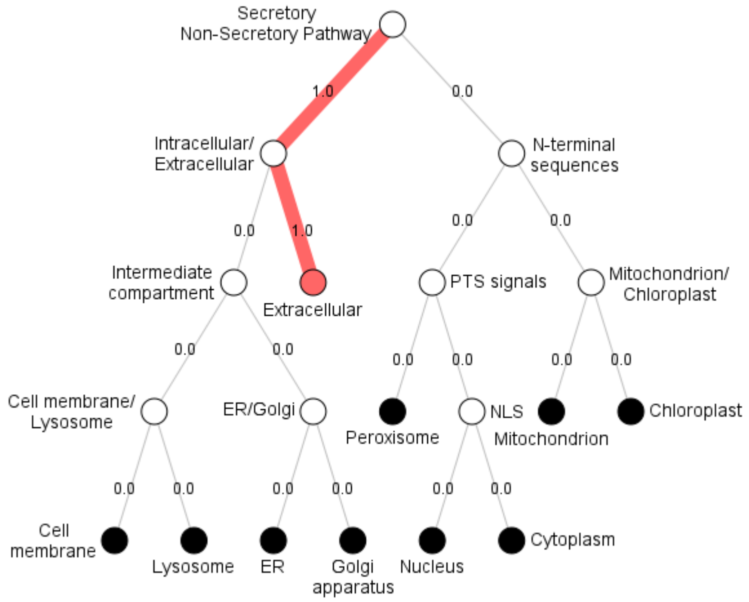

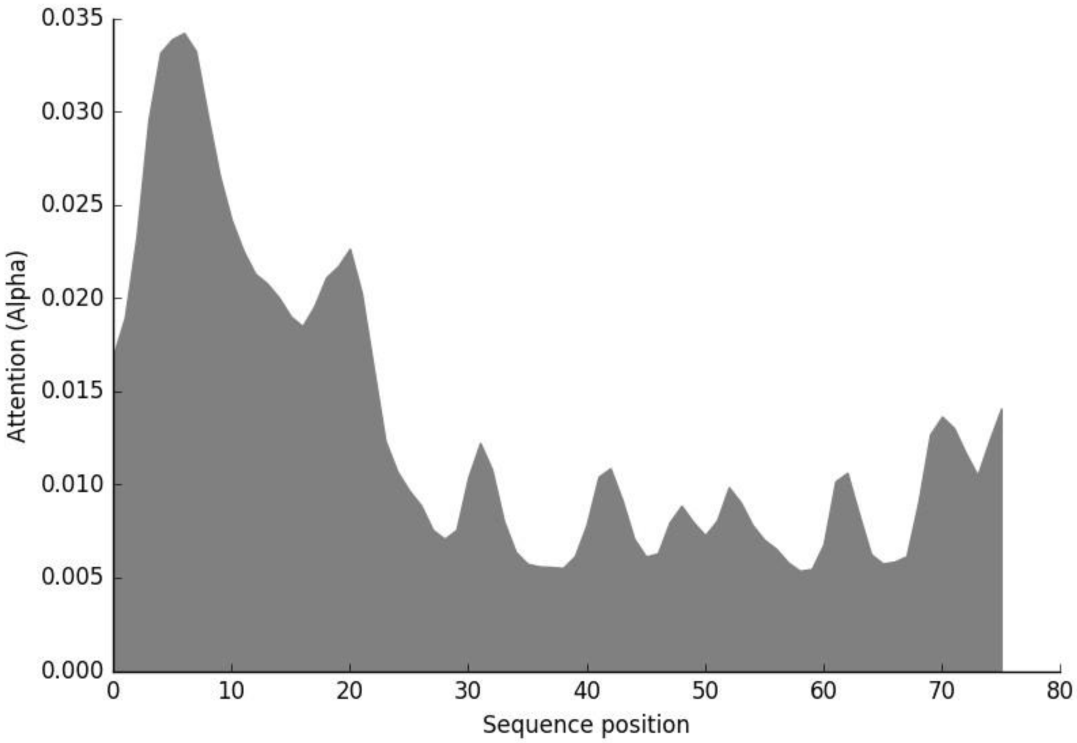

NP_001234872.2
Prediction: Extracellular, Soluble

Localization	Extracellular	Lysosome/Vacuole	Endoplasmic reticulum	Cell membrane	Mitochondrion	Golgi apparatus	Cytoplasm	Plastid	Nucleus	Peroxisome
Likelihood	1	0	0	0	0	0	0	0	0	0

Type	Soluble	Membrane
Likelihood	1	0

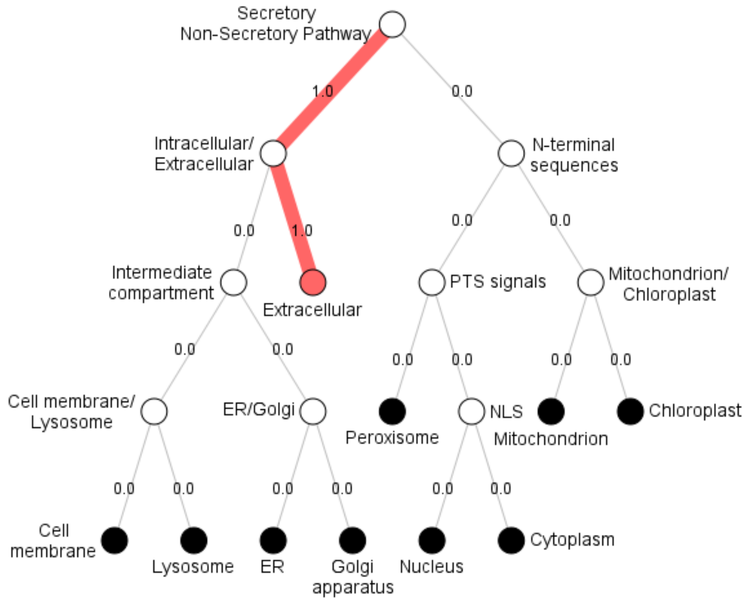

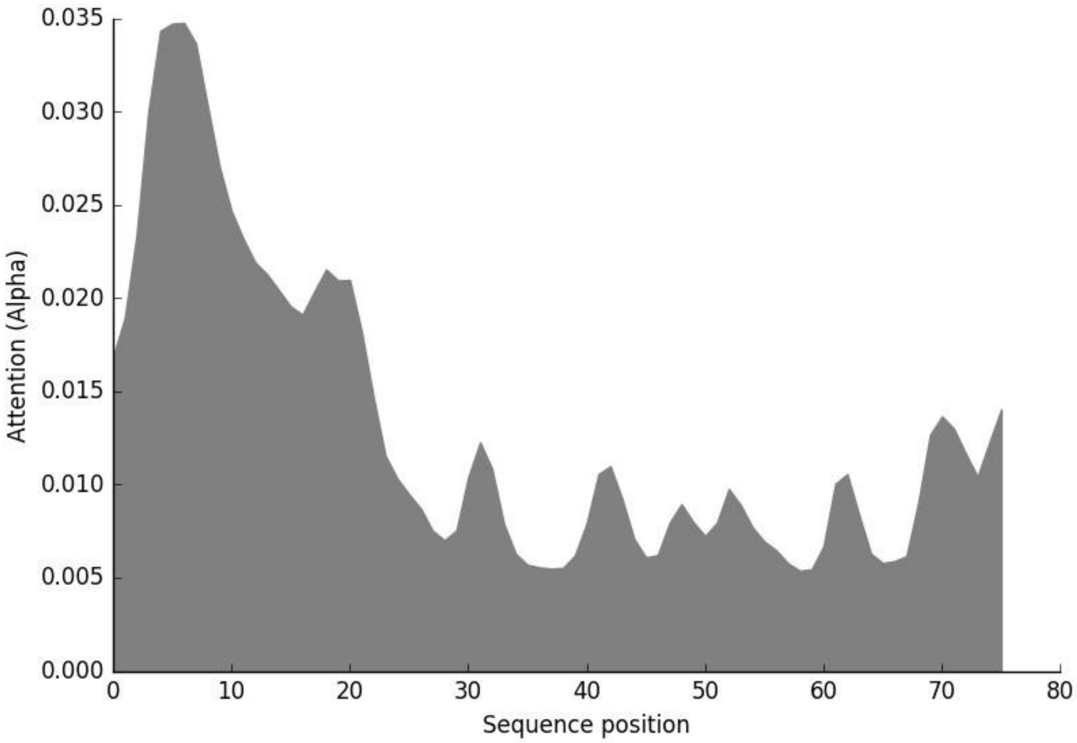

NP_001297247.1
Prediction: Extracellular, Soluble

Localization	Extracellular	Lysosome/Vacuole	Endoplasmic reticulum	Cell membrane	Cytoplasm	Golgi apparatus	Mitochondrion	Plastid	Nucleus	Peroxisome
**Likelihood**	0.9998	0.0001	0	0	0	0	0	0	0	0

Type	Soluble	Membrane
**Likelihood**	1	0

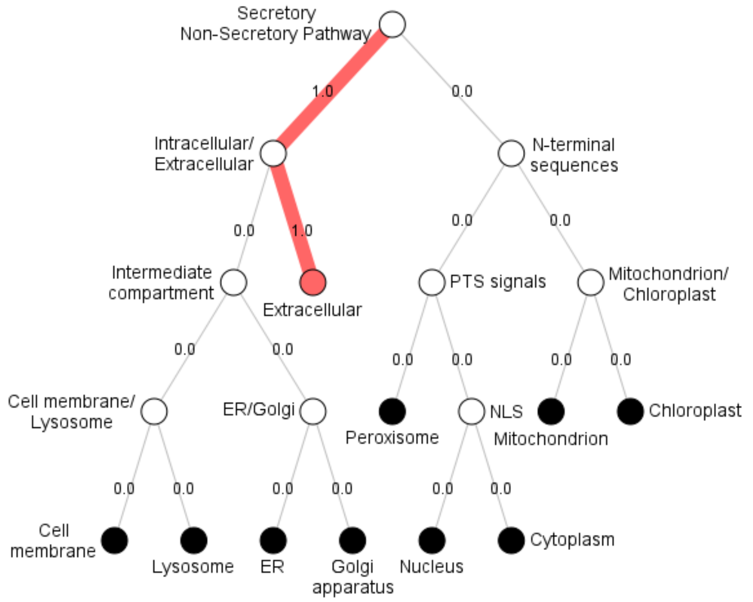

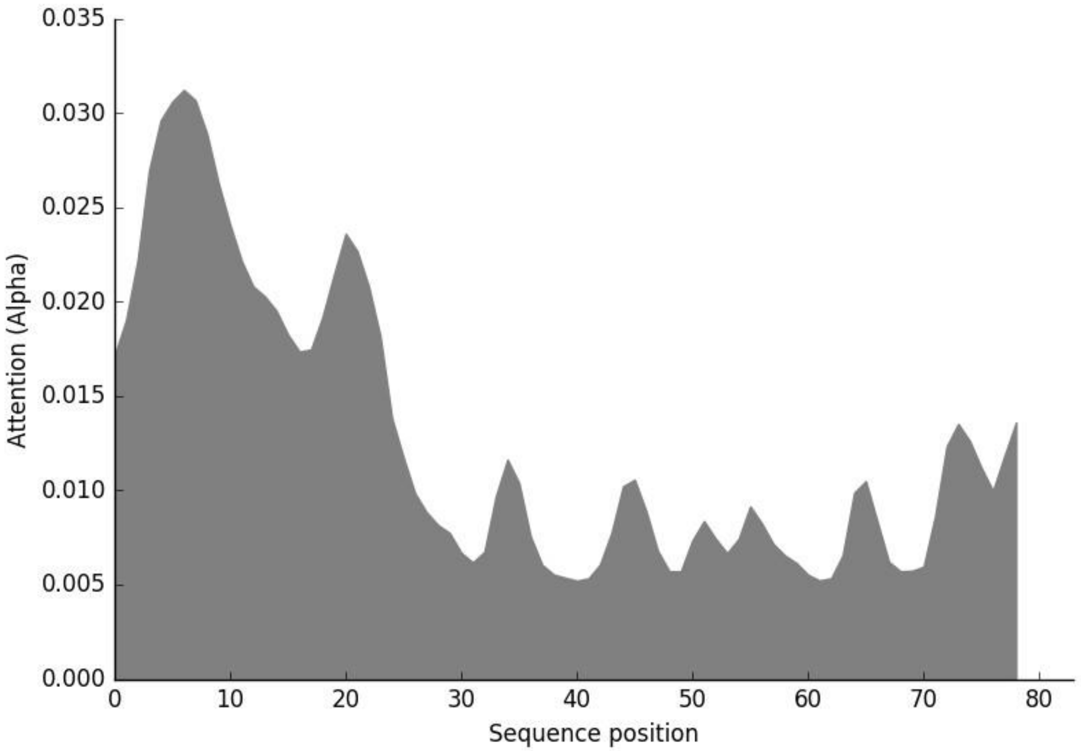

XP_019069532.1
Prediction: Extracellular, Soluble

Localization	Extracellular	Cell membrane	Lysosome/Vacuole	Cytoplasm	Endoplasmic reticulum	Golgi apparatus	Mitochondrion	Plastid	Nucleus	Peroxisome
Likelihood	0.9977	0.0012	0.0011	0	0	0	0	0	0	0

Type	Soluble	Membrane
Likelihood	0.9986	0.0014

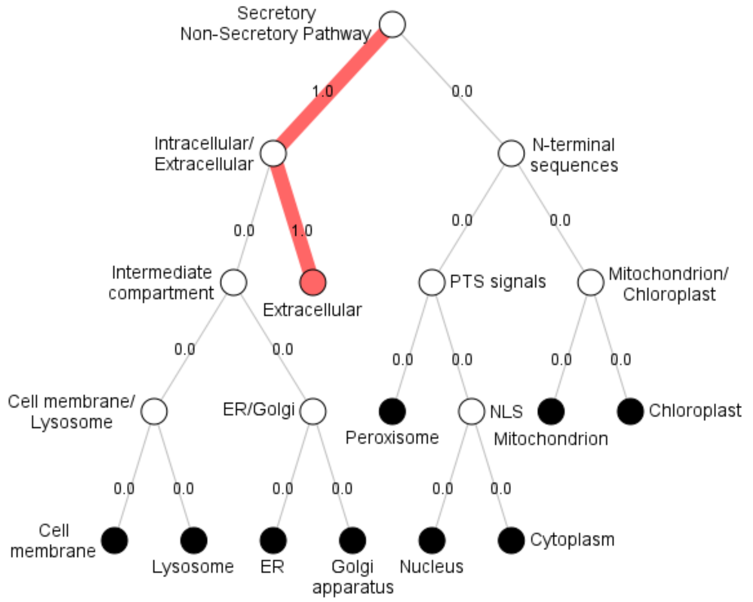

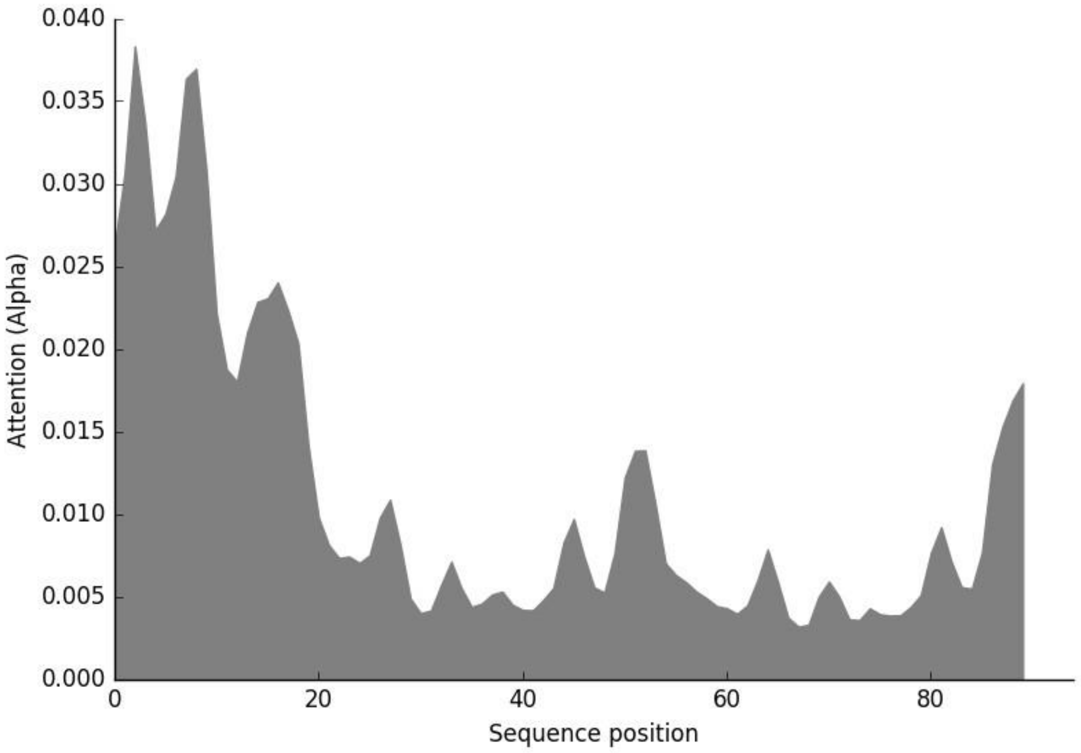

Supplement: Supplementary file 1 [file ijms-21-09380-s001.zip › supporting-information-1.pdf]
