# Supplementary material for: Structural Diversity and Highly Specific Host-Pathogen Transcriptional Regulation of Defensin Genes Is Revealed in Tomato
Source: Int J Mol Sci. 2020 Dec 9;21(24):9380. doi: 10.3390/ijms21249380 (PMC7764197; doi:10.3390/ijms21249380)
Supplement: Supplementary file 1 [file ijms-21-09380-s001.zip › supporting-information-2.pdf]

Structural diversity and highly specific host-pathogen transcriptional regulation of defensin genes is revealed in tomato seedlings

Supporting information 2: Signal peptide prediction for *Solanum lycopersicum* defensin genes

```
# NP_001234872.2 Length: 76
# NP_001234872.2 Number of predicted TMHs:  1
# NP_001234872.2 Exp number of AAs in TMHs: 20.08864
# NP_001234872.2 Exp number, first 60 AAs: 20.08864
# NP_001234872.2 Total prob of N-in:        0.65780
# NP_001234872.2 POSSIBLE N-term signal sequence
NP_001234872.2  TMHMM2.0        inside       1     6
NP_001234872.2  TMHMM2.0        TMhelix      7    29
NP_001234872.2  TMHMM2.0        outside     30    76
```

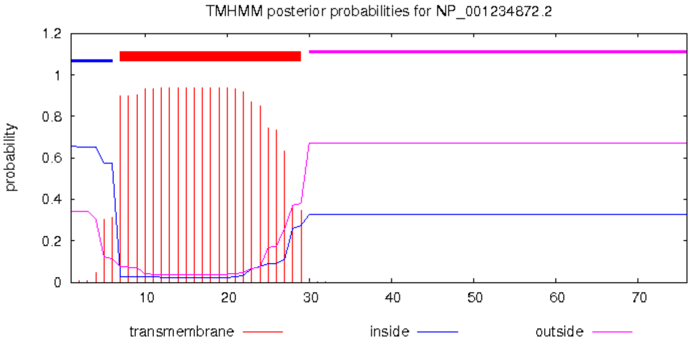


```
# XP_025886757.1 Length: 90
# XP_025886757.1 Number of predicted TMHs:  1
# XP_025886757.1 Exp number of AAs in TMHs: 19.3174
# XP_025886757.1 Exp number, first 60 AAs: 19.31738
# XP_025886757.1 Total prob of N-in:        0.64399
# XP_025886757.1 POSSIBLE N-term signal sequence
XP_025886757.1  TMHMM2.0        inside       1     6
XP_025886757.1  TMHMM2.0        TMhelix      7    25
XP_025886757.1  TMHMM2.0        outside     26    90
```

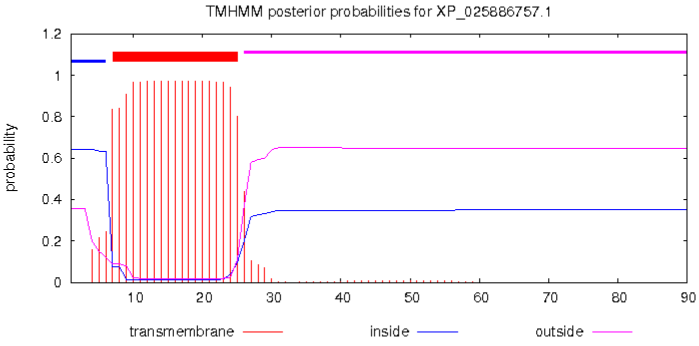


```
# XP_019069532.1 Length: 90
# XP_019069532.1 Number of predicted TMHs:  1
# XP_019069532.1 Exp number of AAs in TMHs: 18.37668
# XP_019069532.1 Exp number, first 60 AAs: 18.37666
# XP_019069532.1 Total prob of N-in:        0.65422
# XP_019069532.1 POSSIBLE N-term signal sequence

XP_019069532.1  TMHMM2.0        inside       1     6
XP_019069532.1  TMHMM2.0        TMhelix      7    25
XP_019069532.1  TMHMM2.0        outside     26    90
```

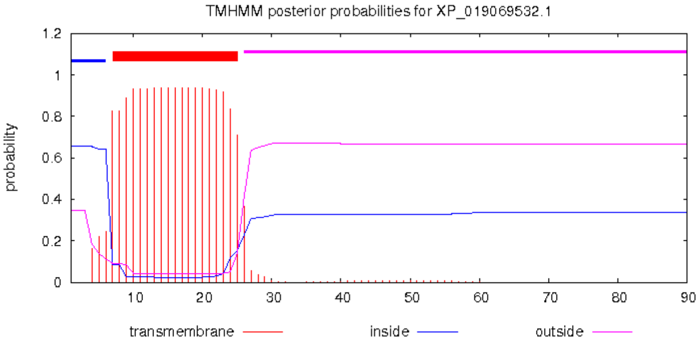


```
# NP_001333453.1 Length: 78
# NP_001333453.1 Number of predicted TMHs:  1
# NP_001333453.1 Exp number of AAs in TMHs: 18.18155
# NP_001333453.1 Exp number, first 60 AAs: 18.18155
# NP_001333453.1 Total prob of N-in:        0.81765
# NP_001333453.1 POSSIBLE N-term signal sequence

NP_001333453.1  TMHMM2.0        inside       1     6
NP_001333453.1  TMHMM2.0        TMhelix      7    29
NP_001333453.1  TMHMM2.0        outside     30    78
```

TMHMM posterior probabilities for NP_001333453.1

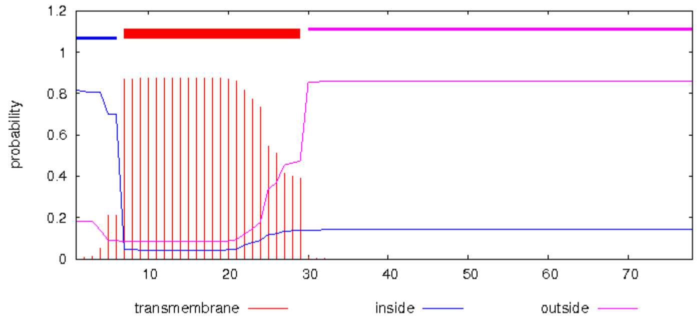


```
# XP_004242851.1 Length: 76
# XP_004242851.1 Number of predicted TMHs:  0
# XP_004242851.1 Exp number of AAs in TMHs: 9.71154
# XP_004242851.1 Exp number, first 60 AAs:  9.70658
# XP_004242851.1 Total prob of N-in:         0.35171
XP_004242851.1  TMHMM2.0        outside      1    76
```

TMHMM posterior probabilities for XP_004242851.1

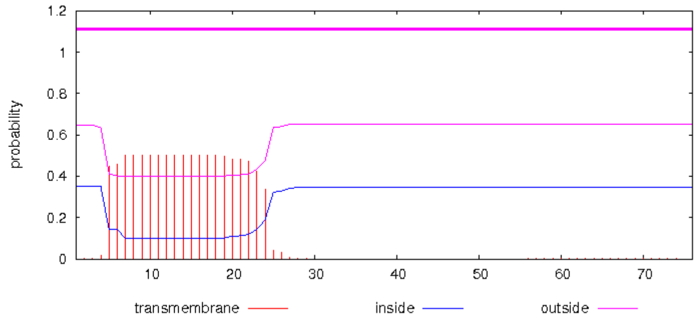


```
# NP_001297246.1 Length: 76
# NP_001297246.1 Number of predicted TMHs:  1
# NP_001297246.1 Exp number of AAs in TMHs: 19.65751
# NP_001297246.1 Exp number, first 60 AAs: 19.6567
# NP_001297246.1 Total prob of N-in:         0.60035
# NP_001297246.1 POSSIBLE N-term signal sequence
NP_001297246.1  TMHMM2.0        outside      1     4
NP_001297246.1  TMHMM2.0        TMhelix      5    27
NP_001297246.1  TMHMM2.0        inside      28    76
```

TMHMM posterior probabilities for NP_001297246.1

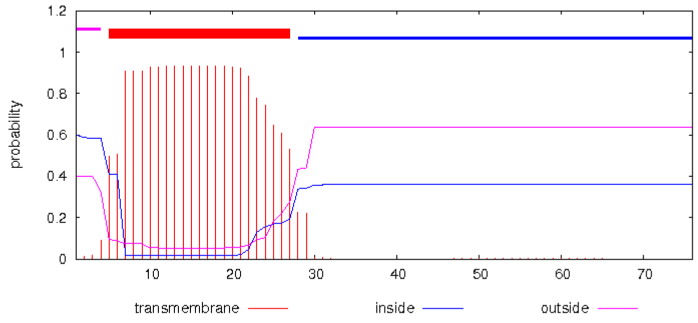


```
# NP_001297247.1 Length: 79
# NP_001297247.1 Number of predicted TMHs:  1
# NP_001297247.1 Exp number of AAs in TMHs: 18.87905
# NP_001297247.1 Exp number, first 60 AAs: 18.87123
# NP_001297247.1 Total prob of N-in:         0.68908
# NP_001297247.1 POSSIBLE N-term signal sequence
NP_001297247.1  TMHMM2.0        outside      1     4
NP_001297247.1  TMHMM2.0        TMhelix      5    27
NP_001297247.1  TMHMM2.0        inside      28    79
```

TMHMM posterior probabilities for NP_001297247.1

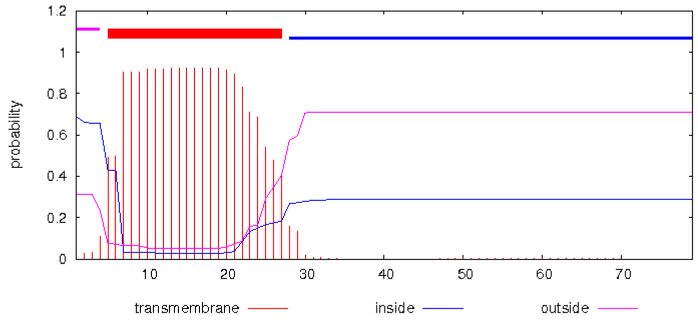


```
# XP_010319372.1 Length: 73
# XP_010319372.1 Number of predicted TMHs:  1
# XP_010319372.1 Exp number of AAs in TMHs: 15.75835
# XP_010319372.1 Exp number, first 60 AAs: 15.75832
# XP_010319372.1 Total prob of N-in:         0.80968
# XP_010319372.1 POSSIBLE N-term signal sequence
```

```
XP_010319372.1  TMHMM2.0        inside       1     6
XP_010319372.1  TMHMM2.0        TMhelix      7    24
XP_010319372.1  TMHMM2.0        outside     25    73
```

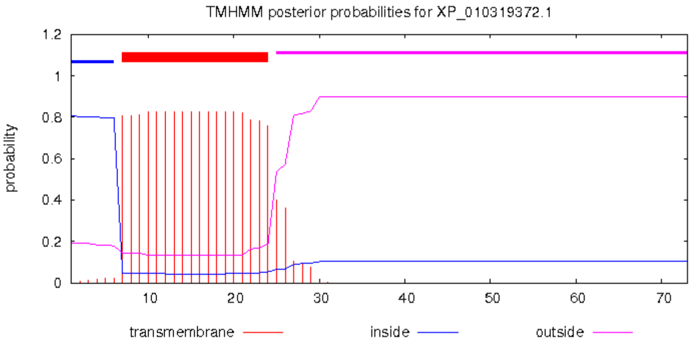

```
# XP_004242838.1 Length: 87
# XP_004242838.1 Number of predicted TMHs:  1
# XP_004242838.1 Exp number of AAs in TMHs: 18.9957
# XP_004242838.1 Exp number, first 60 AAs: 18.99367
# XP_004242838.1 Total prob of N-in:        0.69912
# XP_004242838.1 POSSIBLE N-term signal sequence
XP_004242838.1  TMHMM2.0        inside       1     6
XP_004242838.1  TMHMM2.0        TMhelix      7    25
XP_004242838.1  TMHMM2.0        outside     26    87
```

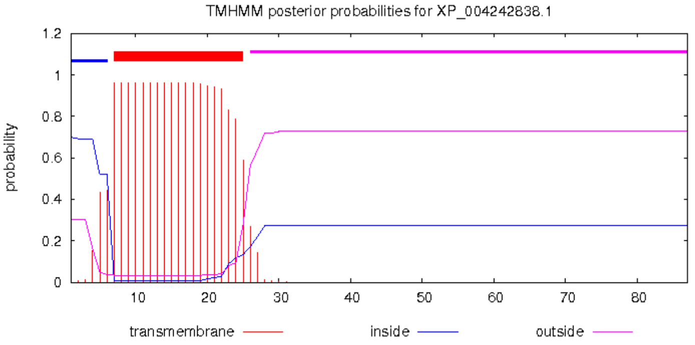

```
# NP_001315592.1 Length: 105
# NP_001315592.1 Number of predicted TMHs:  1
# NP_001315592.1 Exp number of AAs in TMHs: 17.26303
# NP_001315592.1 Exp number, first 60 AAs: 17.26303
# NP_001315592.1 Total prob of N-in:        0.19849
# NP_001315592.1 POSSIBLE N-term signal sequence
NP_001315592.1  TMHMM2.0        outside      1     3
NP_001315592.1  TMHMM2.0        TMhelix      4    21
NP_001315592.1  TMHMM2.0        inside      22   105
```

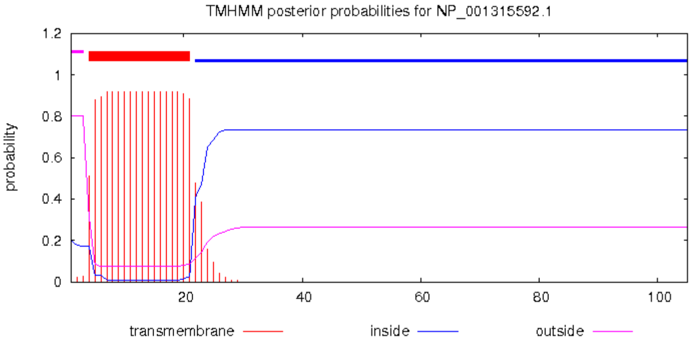
