# Supplementary material for: Structural Diversity and Highly Specific Host-Pathogen Transcriptional Regulation of Defensin Genes Is Revealed in Tomato
Source: Int J Mol Sci. 2020 Dec 9;21(24):9380. doi: 10.3390/ijms21249380 (PMC7764197; doi:10.3390/ijms21249380)

Int. J. Mol. Sci. 2020, 21, x; doi: FOR PEER REVIEW

Nikoloudakis et al.

Structural diversity and highly specific host-pathogen transcriptional regulation of defensin genes is revealed in tomato seedlings

Supporting information 3: Proportion of Amino acids across *Solanum lycopersicum* defensin genes

Amino acids	One letter code	Percentage
Tyr	(Y)	2.50%
Trp	(W)	0.80%
Val	(V)	4.90%
Thr	(T)	6.00%
Ser	(S)	7.20%
Arg	(R)	6.60%
Gln	(Q)	2.80%
Pro	(P)	2.70%
Asn	(N)	3.40%
Met	(M)	3.70%
Leu	(L)	7.20%
Lys	(K)	6.10%
Ile	(I)	4.20%
His	(H)	2.50%
Gly	(G)	7.80%
Phe	(F)	6.50%
Glu	(E)	4.70%
Asp	(D)	2.50%
Cys	(C)	11.10%
Ala	(A)	6.50%

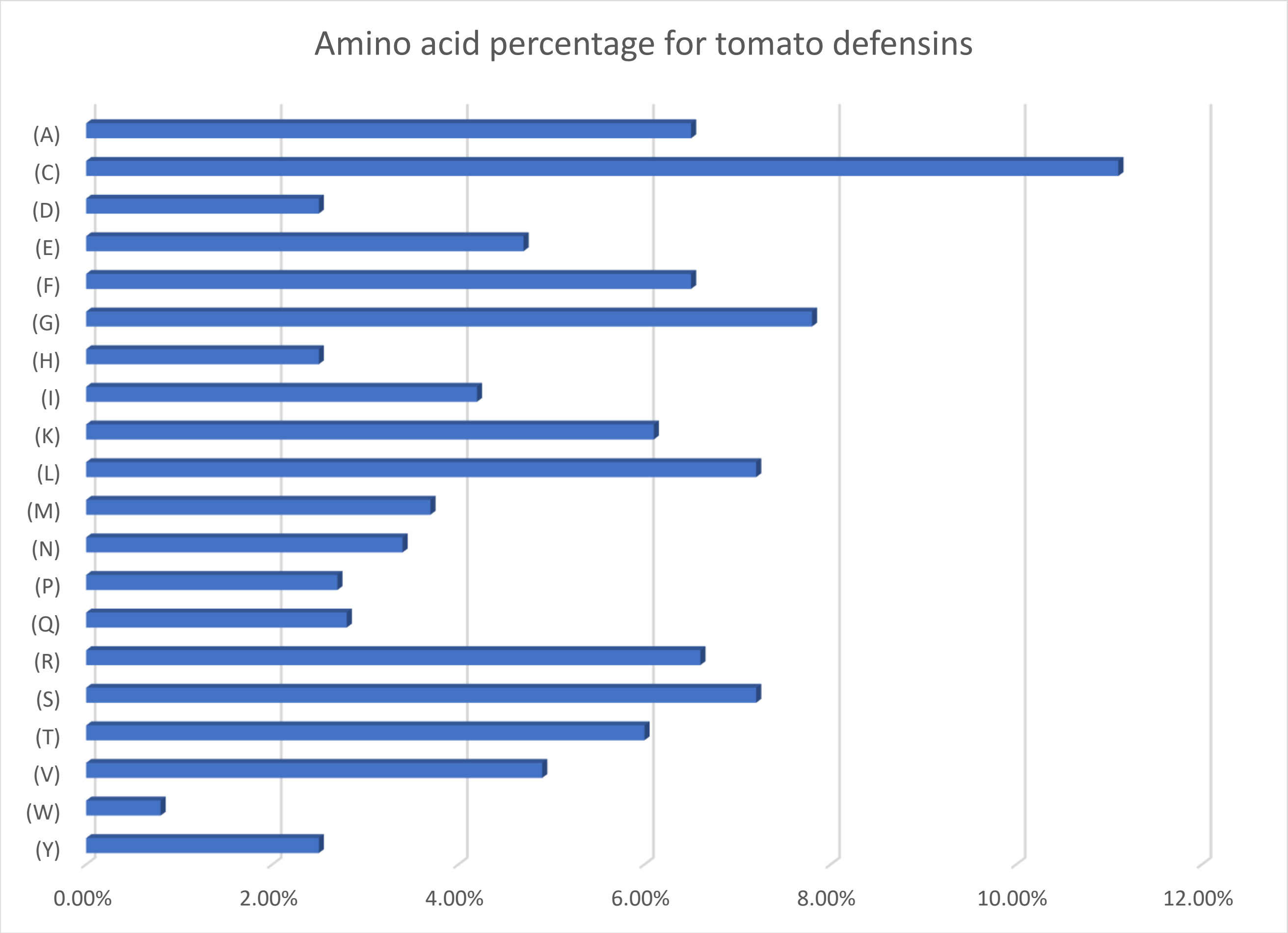

Supplement: Supplementary file 1 [file ijms-21-09380-s001.zip › supporting-information-3.pdf]
