# Supplementary material for: Structural Diversity and Highly Specific Host-Pathogen Transcriptional Regulation of Defensin Genes Is Revealed in Tomato
Source: Int J Mol Sci. 2020 Dec 9;21(24):9380. doi: 10.3390/ijms21249380 (PMC7764197; doi:10.3390/ijms21249380)
Supplement: Supplementary file 1 [file ijms-21-09380-s001.zip › supporting-information-4.pdf]

Int. J. Mol. Sci. 2020, 21, x; doi: FOR PEER REVIEW

Nikoloudakis et al. Structural diversity and highly specific host-pathogen transcriptional regulation of defensin genes is revealed in tomato seedlings

Supporting information 4: Phylogenetic relationships across defensin genes

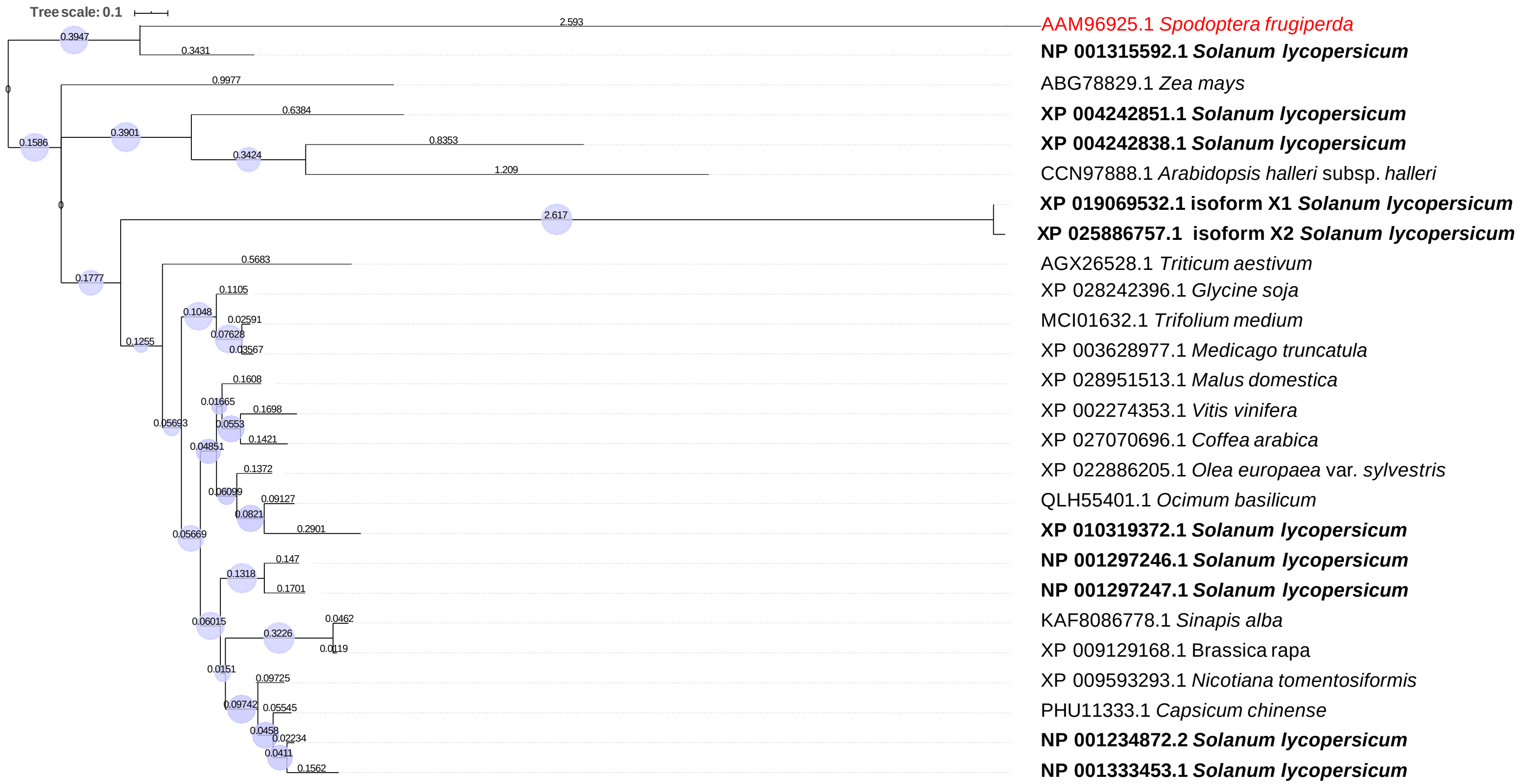

Defensin proteins were align using the MUSCLE alignment algorithm and phylogenetic coalescences were calculated with PhyML (Maximum likelihood). Branch length (numbers) and bootstrap values (circles) are depicted across groups. With bold font are the tomato defensins analyzed in the current study. Fall armyworm (*Spodoptera frugiperda*) was employed as an outgroup. The iTOL server (https://itol.embl.de/) was used for tree displaying
