# Supplementary material for: Structural Diversity and Highly Specific Host-Pathogen Transcriptional Regulation of Defensin Genes Is Revealed in Tomato
Source: Int J Mol Sci. 2020 Dec 9;21(24):9380. doi: 10.3390/ijms21249380 (PMC7764197; doi:10.3390/ijms21249380)

Int. J. Mol. Sci. 2020, 21, x; doi: FOR PEER REVIEW

Nikoloudakis et al.

Structural diversity and highly specific host-pathogen transcriptional regulation of defensin genes is revealed in tomato seedlings

Supporting information 5: Stacked sequence profiles of tomato defensin accessions.

>0001

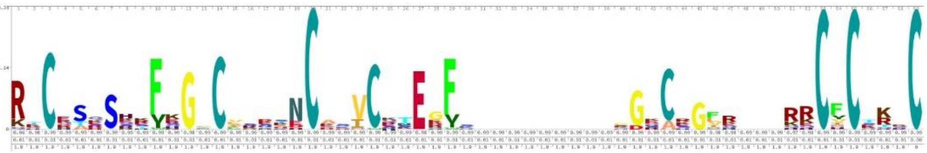

>0002

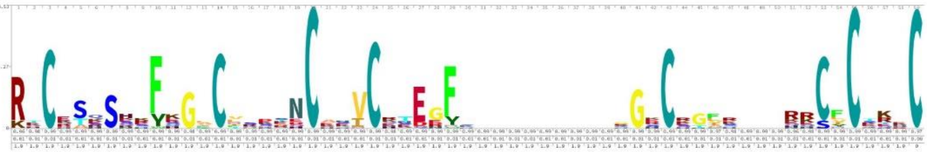

>0003

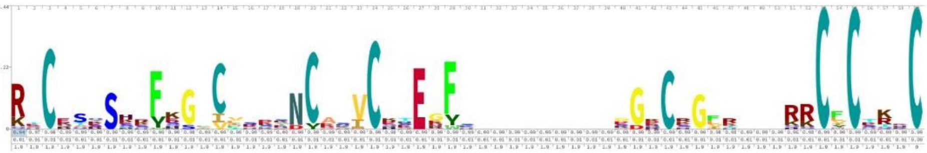

>0004

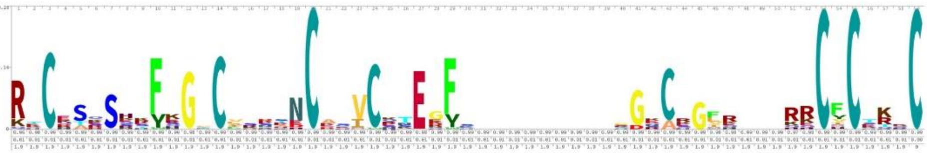

>0005

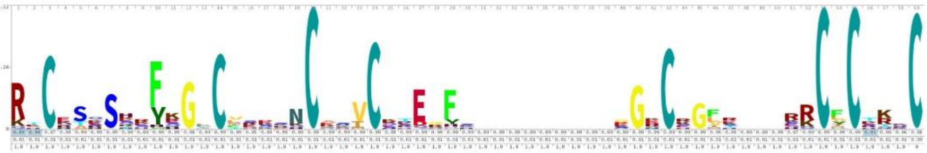

>0006

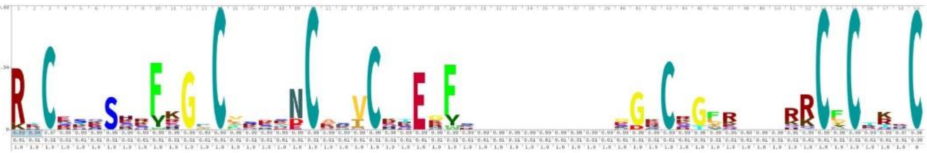

>0007

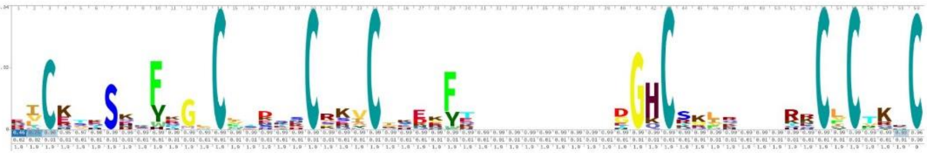

>0008

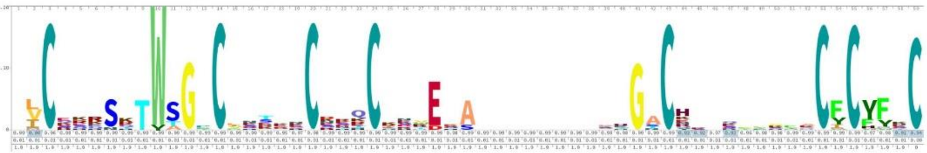

>0009

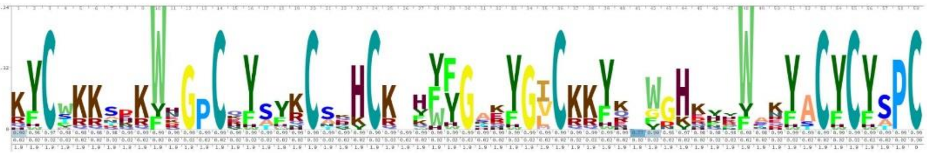

Supplement: Supplementary file 1 [file ijms-21-09380-s001.zip › supporting-information-5.pdf]
