# Supplementary material for: Structural Diversity and Highly Specific Host-Pathogen Transcriptional Regulation of Defensin Genes Is Revealed in Tomato
Source: Int J Mol Sci. 2020 Dec 9;21(24):9380. doi: 10.3390/ijms21249380 (PMC7764197; doi:10.3390/ijms21249380)

Int. J. Mol. Sci. 2020, 21, x; doi: FOR PEER REVIEW
Nikoloudakis et al.
Structural diversity and highly specific host-pathogen transcriptional regulation of defensin genes is revealed in tomato seedlings

Supporting information 6: Relative transcription for tomato defensin genes exposed to discrete biotic/abiotic stresses. Relative mRNA abundance was assessed by real-time RT-qPCR. Data were standardized as reported in the Material and Methods segment. Significant differences between treatments were determined by ANOVA and post-hoc comparisons by least significant difference (P < 0.05). Bars represent means (± SE) of three biological replications. Vertical axis shows the relative expression to the control gene Ubiquitin.

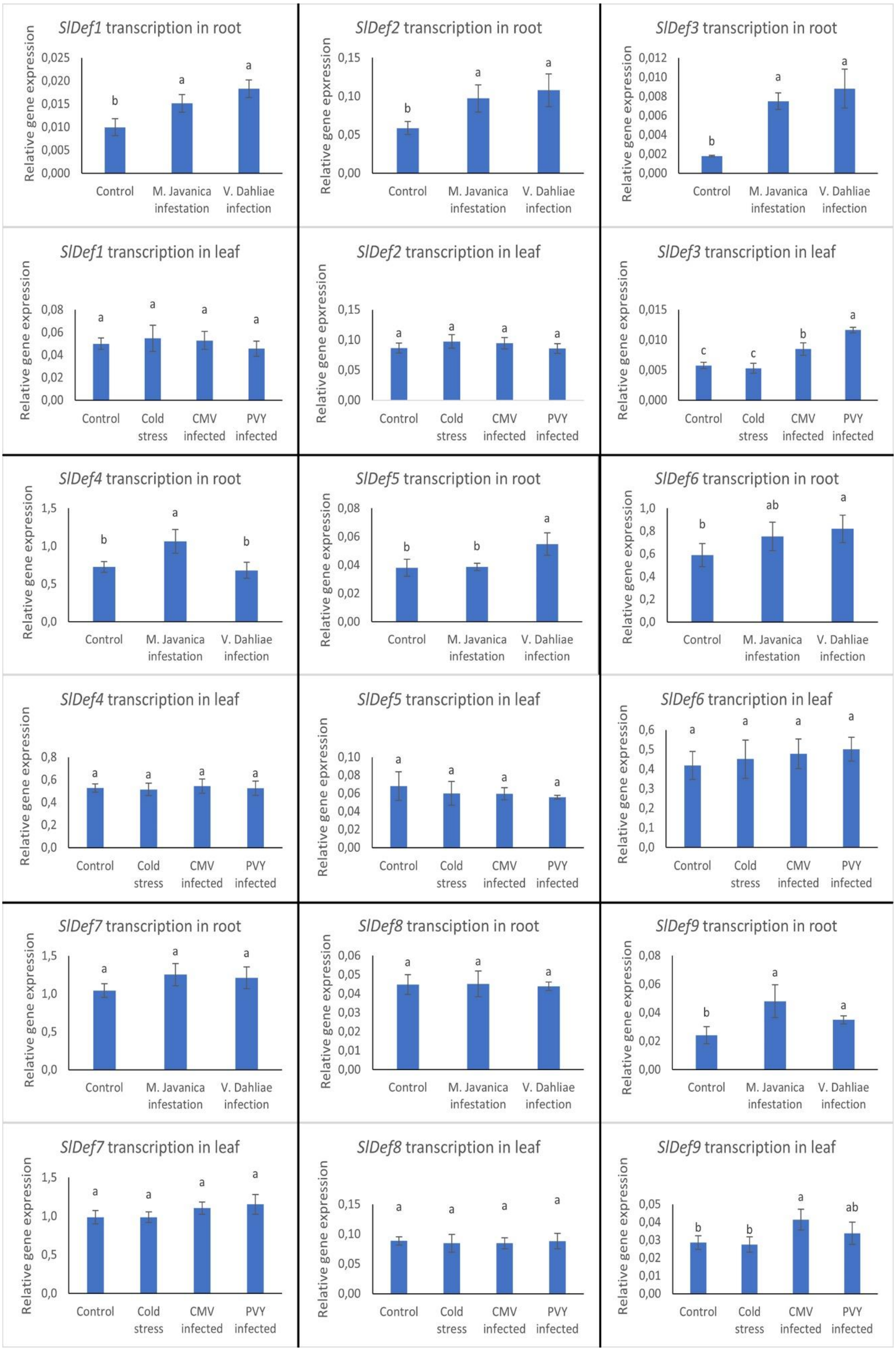

Supplement: Supplementary file 1 [file ijms-21-09380-s001.zip › supporting-information-6.pdf]
