# Supplementary material for: Structural Diversity and Highly Specific Host-Pathogen Transcriptional Regulation of Defensin Genes Is Revealed in Tomato
Source: Int J Mol Sci. 2020 Dec 9;21(24):9380. doi: 10.3390/ijms21249380 (PMC7764197; doi:10.3390/ijms21249380)
Supplement: Supplementary file 1 [file ijms-21-09380-s001.zip › supporting-information-7.pdf]

Int. J. Mol. Sci. 2020, 21, x; doi: FOR PEER REVIEW

Nikoloudakis et al.

Structural diversity and highly specific host-pathogen transcriptional regulation of defensin genes is revealed in tomato seedlings

Supporting information 7: Primers used for the amplification of *Solanum lycopersicum* defensin genes (S1Def1 - S1Def9) across treatments, and Ubiquitin (UBQ) control

	**Gene code**	**Forward primer (5'-3')**	**Reverse primer (5'-3')**
1	S1Def1	AGGACCATGTGTGAGTGAGA	CGACAATCACCACCGGAAAA
2	S1Def2	TTTCATGTGTGATAGGGCGTGA	GGCAACCCTTGATCGCATGT
3	S1Def3	AGCACCAAGCCAAACTTTCC	TTTGCTTCCTCACCCAAAGT
4	S1Def4	TGGTTTTCTTGCCCTCCTCA	GTGGCAAGGTGAGTAGCAGT
5	S1Def5	CCGTGCGTGAGGAAGAACAA	GTTTAGCGCAGAAGCAACGG
6	S1Def6	TTTTGCTTCCTCCTCGTTGC	ATGTCTTGCTCCGCCTTTGA
7	S1Def7	AATTGTGGTTCCGTTTGCCG	CAATTCCGGGTGCAAAAGCA
8	S1Def8	TTAGCAGTGCAGAGGCAAGA	CTTCAGTCTGGCAAACGGTG
9	S1Def9	ACTGAAATGGGACCAACGAGAA	TGGTCCCTTGAAACGATGGC
10	UBQ	GCAGACTATAACATCCAGAAAGAG	AACAACAAAGCACACAGCCATC
